# Fabp5 is a common gene between a high-cholesterol diet and acute pancreatitis

**DOI:** 10.3389/fnut.2023.1284985

**Published:** 2023-12-22

**Authors:** Minhao Qiu, Fangfang Cai, Yining Huang, Liang Sun, Jianmin Li, Wei Wang, Zarrin Basharat, Maddalena Zippi, Hemant Goyal, Jingye Pan, Wandong Hong

**Affiliations:** ^1^Department of Gastroenterology and Hepatology, The First Affiliated Hospital of Wenzhou Medical University, Wenzhou, China; ^2^School of Mental Health, Wenzhou Medical University, Wenzhou, China; ^3^Department of Pathology, The First Affiliated Hospital of Wenzhou Medical University, Wenzhou, China; ^4^Alpha Genomics (Private) Limited, Rawalpindi, Pakistan; ^5^Unit of Gastroenterology and Digestive Endoscopy, Sandro Pertini Hospital, Rome, Italy; ^6^Borland Groover Clinic, Baptist Medical Center, Jacksonville, FL, United States; ^7^Intensive Care Unit, The First Affiliated Hospital of Wenzhou Medical University, Wenzhou, China

**Keywords:** hypercholesterolemia, acute pancreatitis, differentially expressed gene, bioinformatic analysis, microarray

## Abstract

**Background and aims:**

Hypercholesterolemia has been identified as risk factor for severe acute pancreatitis (AP). We aimed to identify the common differentially expressed genes (DEGs) between a high-cholesterol diet and AP.

**Methods:**

We retrived gene expression profiles from the GEO database. DEGs were assessed using GEO2R. For AP hub genes, we conducted functional enrichment analysis and protein–protein interaction (PPI) analysis. GeneMANIA and correlation analysis were employed to predict potential DEG mechanisms. Validation was done across various healthy human tissues, pancreatic adenocarcinoma, peripheral blood in AP patients, and Sprague–Dawley rats with AP.

**Results:**

The gene “Fabp5” emerged as the sole common DEG shared by a high-cholesterol diet and AP. Using the 12 topological analysis methods in PPI network analysis, Rela, Actb, Cdh1, and Vcl were identified as hub DEGs. GeneMANIA revealed 77.6% physical interactions among Fabp5, TLR4, and Rela, while genetic correlation analysis indicated moderate associations among them. Peripheral blood analysis yielded area under the ROC curve (AUC) values of 0.71, 0.63, 0.74, 0.64, and 0.91 for Fabp5, TLR4, Actb, Cdh1 genes, and artificial neural network (ANN) model respectively, in predicting severe AP. *In vivo* immunohistochemical analysis demonstrated higher Fabp5 expression in the hyperlipidemia-associated AP group compared to the AP and control groups.

**Conclusion:**

Fabp5 emerged as the common DEG connecting a high-cholesterol diet and AP. Rela was highlighted as a crucial hub gene in AP. Genetic interactions were observed among Fabp5, TLR4, and Rela. An ANN model consisting of Fabp5, TLR4, Actb, and Cdh1 was helpful in predicting severe AP.

## Highlights

- “Fabp5” gene was identified as the common differentially expressed gene between a high-cholesterol diet and acute pancreatitis.
- Rela, Actb, Cdh1, and Vcl were hub genes of acute pancreatitis.
- High-cholesterol diet may affect the severity of acute pancreatitis by regulating the expression of Fabp5, resulting in enhanced activation of TLR signaling and the Nuclear Factor-κB pathway.
- An artificial neural network (ANN) model consisting of Fabp5, TLR4, Actb and Cdh1 was useful in predicting severe acute pancreatitis.

## 1 Introduction

Acute pancreatitis (AP) is a common gastrointestinal disorder with marked variation in severity. In most patients, AP has a self-limiting and mild course (1). However, a subset of patients (10–20%) might progress to severe acute pancreatitis (SAP) with high mortality (2). Therefore, early identification of patients who are at high risk for developing the systemic complications and organ failure may be beneficial in selecting individuals who would derive the most benefit from early interventions (3). However, the existing scoring systems such as Bedside Index of Severity in Acute Pancreatitis (BISAP) have moderate accuracy in predicting the severity of AP (1). It was suggested that complex gene–environment interactions may partly explain both diverse clinical appearance and a sometimes hard-to-predict the course of progression (4). However, curently no predictive genetic biomarkers have consistently shown usefulness for implementation in the routine clinical care of AP patients (4).

The rapid development of bioinformatics approaches is aiding in gaining a deeper understanding of disease pathobiology at the genetic level and in the development of predictive genetic biomarkers. Numerous studies have explored potential hub differentially expressed genes (DEGs) for acute pancreatitis through bioinformatics analysis (5–8). Fan et al. have suggested that genes EGFR, CDH1, ACTB, CD44, and VCL may play vital role in the pathogenesis of AP (5). Ji et al. suggested that genes CDH1 and CLDN4 play a key role in the pathogenesis of AP (6). Zhong et al. reported that both gene CDH1 and VCL were core genes in AP (7). Zhang et al. suggested that AP and hypertriglyceridemia shared common DEGs, WSB1 and FBXO4 (8). Therefore, different studies reported different hub DEGs for AP, which might be due to implementation of different bioinformatic analysis methods (5–8). In addition, these studies did not evaluate test hub genes as predictive factors of SAP.

Hypercholesterolemia, mainly caused by a high-cholesterol diet, has been identified as a risk factor of SAP (9). Patients with hypercholesterolemia (>240 mg/dL) had a significantly higher incidence of SAP and protracted hospital stays when compared to moderate total cholesterol levels (160–240 mg/dL) (1). Czako et al. suggested that hyperlipidemia induced by a cholesterol-rich diet could aggravate the severity of necrotizing pancreatitis by nuclear factor-kappa B (NF-κB) activation in rats (10). In addition, the accumulation of cholesterol promotes the activation of both innate and adaptive inflammatory responses, including the augmentation of Toll-like receptor 4 (TLR4) signaling (9), which plays a significant proinflammatory role in the progression of AP (11). Therefore, both TLR4 signaling and NF-κB may play a role in the pathogenesis of high-cholesterol diet associated AP. Despite these potential molecular mechanisms, the genomic relationship between a high-cholesterol diet and AP has not been investigated thus far.

Therefore, the primary aim of this study was to identify the common DEGs between a high-cholesterol diet and AP as well as its possible relationship with TLR4 signaling and NF-κB. The second aim of this study was to identify hub DEGs for AP and its possible role in predicting SAP.

## 2 Methods

### 2.1 Data source and DEG identification

Five datasets (Accession ID: GSE3644/GSE65146/GSE109227/GSE159656/GSE194331) were downloaded from the Gene Expression Omnibus (GEO) database and further processed by the online tool GEO2R.^1^ The common over-lapping mRNAs among GSE3644, GSE65146, and GSE109227 dataset were identified as AP-related DEGs. The common over-lapping mRNAs among GSE3644, GSE65146, GSE109227, and GSE159656 datasets were identified as common DEGs between the high-cholesterol diet and AP.

The dataset GSE194331 was used to validate the role of the common DEGs in peripheral blood in patients with AP. It was gene expression profiling of AP by high throughput sequencing (12). In this dataset, peripheral blood was collected from 87 patients with AP of varying severity (mild = 57, moderate–severe = 20, severe = 10) within 24 h of presentation to the hospital and from 32 healthy controls (12). The details of selected microarray datasets are shown in Supplementary Tables S1, S2.

DEGs were screened from the GEO database with the threshold of |log2FC| >1 and adjusted *p* < 0.05. Of these, the genes in the common datasets with logFC >0 were considered upregulated, while those with logFC <0 were considered down-regulated.

### 2.2 Functional enrichment analysis

Gene ontology (GO) is a systematical approach to gene annotation, RNA, and protein expression. The Kyoto Encyclopedia of Genes and Genome (KEGG) is an online database of genomes, enzymatic pathways, and biochemicals. The Database for Annotation, Visualization and Integrated Discovery (DAVID) is a bioinformation database^2^ that integrates biological data and analysis tools to help extract systematic biological function information for gene or protein lists. In this study, the DAVID database was used to perform GO analysis and KEGG pathway enrichment analysis, aiding in the classification of DEGs (*p* value <0.05) (13).

### 2.3 The protein–protein interaction (PPI) network construction and hub genes

The Search Tool for the Retrieval of Interacting Genes (STRING^3^) online database was utilized to explore the functional physical interactions between DEG-encoded proteins. These interactions represent complex biological functions, and were used to construct a PPI network (or graph), where proteins serve as nodes, and interactions between them form edges. This network was then studied to unveil complex pathways and reveal the functions of unknown proteins (14). PPI networks are mathematical representations of physical contacts between proteins in the cell. Perturbations in the PPI network that affect essential proteins can lead to disorders in specific cellular functions (15). PPI pairs with a combined score > 0.4 were mapped into the network. Hub genes were identified using the cytoHubba plug-in of Cytoscape software, which ranks nodes in a network based on their features and identifies hub objects and sub-networks from the interactome (16). It provides 12 topological analysis methods, including Edge Percolated Component, Degree, Maximum Neighborhood Component, Density of Maximum Neighborhood Component, Maximal Clique Centrality, Clustering Coefficient and six centralities (Bottleneck, EcCentricity, Closeness, Radiality, Betweenness, and Stress) based on shortest paths (16). The frequencies of occurrence of each gene selected in the 12 topological analysis methods were noted. If genes were selected by 75% (9/12) or more of the methods, they were considered hub genes.

### 2.4 Prediction of potential mechanism and correlation analysis

GeneMANIA^4^ was used to predict the potential mechanism for the common DEGs between the high-cholesterol diet and AP. It is a flexible, user-friendly website to generate hypotheses about gene function, analyzing gene lists and prioritizing genes for functional assays (17). GeneMANIA has the capability to expand the user’s gene list by incorporating genes that are functionally similar or share properties with the initial query genes. It assigns weights to each functional genomic dataset based on its predictive value for the query, and then presents an interactive functional association network. This network effectively illustrates the relationships among the genes and the dataset, providing valuable insights into their functional associations (18). Correlation among genes was evaluated via Gene Expression Profiling Interactive Analysis (GEPIA) database^5^ (19). GEPIA is an online tool, based on The Cancer Genome Atlas (TCGA) and Genotype-Tissue Expression (GTEx^6^). It provides large-scale function analysis, including profiling plotting, DEG mapping, correlation analysis, patient survival analysis, similar gene detection, and dimensionality reduction analysis. In this study, we explored the relationship among expression values of different genes in individuals with healthy pancreas and pancreatic adenocarcinoma, through Pearson correlation analysis.

### 2.5 Validation in different healthy human tissues and pancreatic adenocarcinoma

The data of the GTEx project was used to validate the hub genes expression in different healthy human tissues. It is an open-access database containing gene expression levels in 328 healthy human pancreas tissues. The prognostic role of AP related hub DEGs in pancreatic adenocarcinoma was evaluated by TCGA project of the GEPIA database (19).

### 2.6 Validation and statistical analysis of peripheral blood in AP patients

As mentioned before, the dataset GSE194331 was used to validate the role of the DEGs in peripheral blood in patients with AP. Nomal distribution was evaluated for the continuous data by Shapiro–Wilk test. Continuous variables were expressed as mean standard deviation (SD) or median and interquartile range (IQR) and compared using the independent-samples *t*-test or Kruskal-Wallis nonparametric test (20). All the variables determined to be different between patients with and without AP through a univariate analysis were further analyzed in patients with and without SAP.

A three-layered feed-forward artificial neural network (ANN) model with a backpropagation algorithm was constructed (21). The ANN model was trained with a maximum of 500 iterations and ten tours (21). The overfit penalty was assigned as 0.001, and the convergence criterion was 0.00001 (21). Five-fold cross-validation was used. The area under the receiver operating characteristic curve (ROC) curve (AUC) was used to evaluate prediction performance. A variable with an AUC above 0.7 was considered significant (3). The sensitivity, specificity, and diagnostic accuracy were calculated, and the best Youden Index (sensitivity+specificity−1) value was used to determine the best cutoff point to predict SAP.

### 2.7 Validation in experimental animal model of AP

Adult male Sprague–Dawley rats (weighing 200–220 g) were acquired from the Animal Centre of Wenzhou Medical University (Wenzhou, China) and were kept in a temperature-controlled room, fed freely. These rats were randomly divided into 3 groups: (1) Control group (CON group): In this group, rats were normally kept for 14 days without any other treatment. (2) AP group: Rats in this group were kept for 14 days with intraperitoneal injections of L-arginine (L-Arg, 2.5 g/kg, twice, 1 h interval) to induce pancreatitis. (3) Hyperlipidemic acute pancreatitis group (HAP group): Rats in this group were injected with poloxamer 407 (P-407, 0.25 g/kg) for 14 days and then given two intermittent injections of L-Arg (2.5 g/kg, twice, 1 h interval) to induce pancreatitis (22–24). After 24 h of the last injection, pancreatic tissues were collected from the abdominal cavity. The tissue was used for Hematoxylin–Eosin (HE) and immunohistochemical staining. These rats were humanely treated as as per the United States (US) National Institutes of Health (NIH) guidelines. The Wenzhou Medical University Animal Policy and Welfare Committee sanctioned all animal care and experimental procedures.

Pancreatic tissues were immediately soaked in 10% formalin for 12 h and was later embedded in paraffin and sectioned (5 μm size). The tissue sections were stained with Hematoxylin–Eosin (HE) and observed under a microscope for pancreatic pathological scores. The pancreatic histopathological scoring criteria were based on the study of Schmidt et al. (25). Multiple randomly selected microscopic fields from at least three rats per group were blindly assessed by a senior pathologist (Professor Jianmin Li, with a work experience of 30 years). The pancreatic histopathological scores, comprising edema, acinar necrosis, hemorrhage and fat necrosis, inflammation and perivascular infiltrate, were statistically analyzed by an independent sample *t*-test. Dewaxing, hydration, and thermal repair were performed, and then the primary antibodies (Fabp5, 12348-1-AP, Proteintech, 1:200) were added and incubated overnight at 4°C. After incubating the tissues with enhanced enzyme-labeled goat anti-rabbit IgG polymer (PV9001, ZSGB-BIO) for 20 min twice at 37°C, the signal was detected using Diaminobenzidine (DAB, DA1010, Solarbio). After statistical analysis, the expression of hub genes was measured using Image-Pro Plus 6.0 and visualized using GraphPad Prism 8.0 (GraphPad Software Inc., United States). All data were presented as means ± SD and *p* value <0.05 was considered statistically significant.

## 3 Results

### 3.1 Identification of DEGs

**Figure 1:**
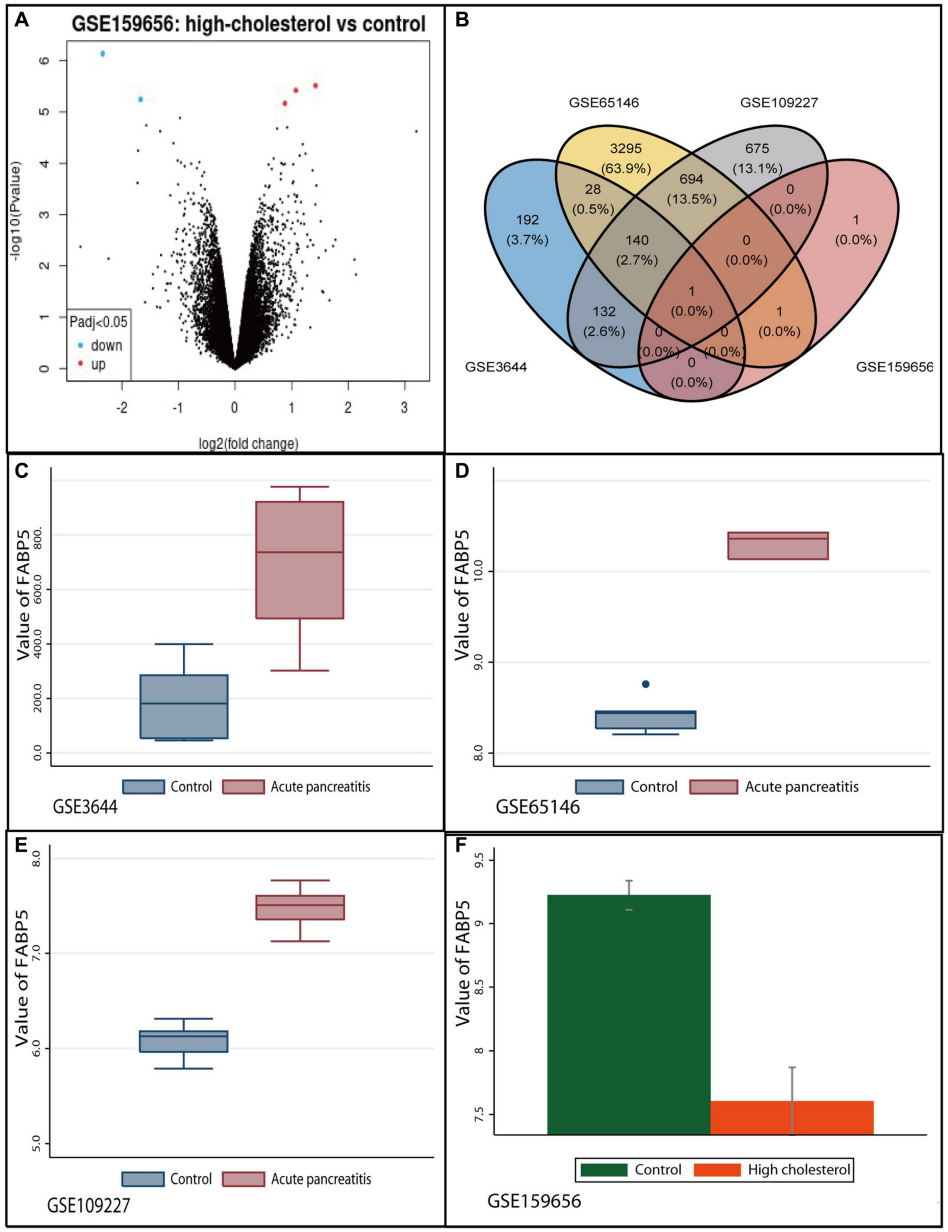
Identification of common differentially expressed gene between high-cholesterol diet and acute pancreatitis. **(A)** Volcano plot of differentially expressed genes in GSE159656 microarrays. Red plot indicates genes with high levels of expression, green plot indicates genes with low levels of expression, and black indicates plot genes with no differentially of expression based on the criterion of *p* value <0.05 and |logFC| > 1.0, respectively. **(B)** The Venn diagram of overlapping genes differentially expressed in four datasets. **(C)** The expression levels of Fabp5 in acute pancreatitis and control groups in GSE3644 microarrays. **(D)** The expression levels of Fabp5 in acute pancreatitis and control groups in GSE65146 microarrays. **(E)** The expression levels of Fabp5 in acute pancreatitis and control groups in GSE109227 microarrays. **(F)** The expression levels of Fabp5 in high cholesterol diet and control groups in GSE159656 microarrays.

There were 141 common AP-related DEGs in the three datasets (Accession ID: GSE3644, GSE65146 and GSE109227) (Supplementary Figure S1). Of these, 130 were upregulated and 11 were down-regulated and regarded as AP-related DEGs (Supplementary Table S3). Five hypercholesterolemia-related DEGs in microarray datasets (GSE159656), of which three were upregulated, and two were down-regulated (Figure 1).

Only the “Fabp5” gene was identified as the overlapping mRNAs among GSE3644, GSE65146, GSE109227, and GSE159656. It was identified as the common DEG between high-cholesterol diet and AP (Figure 1). The relevant protein was “fatty acid binding protein 5, epidermal” for the “Fabp5” gene. We found that “Fabp5” gene was upregulated in AP but down-regulated in high-cholesterol diet compared to the control (Figure 1).

### 3.2 Gene ontology and KEGG pathway analysis of AP-related DEGs

GO term enrichment analysis was performed to investigate the functional annotation of the screened DEGs through the DAVID database. Three categories of GO terms, including Biological Process (BP), Cellular Component (CC), and Molecular Function (MF) results were mined, and are presented in Supplementary Figures S2, S3. The color of the bubble indicates the *p*-value of the term, and the bubble size has a positive relation with the number of the DEGs involved in the term.

DEG-enriched GO terms in the BP mainly covered angiogenesis, positive regulation of macroautophagy, cell migration,transcription, DNA-templated, angiogenesis, and negative regulation of extrinsic apoptotic signaling pathway, platelet aggregation, response to insulin, cell migration, response to oxidative stress, response to hypoxia, sarcomere organization, positive regulation of transcription from RNA polymerase II promoter etc. (Supplementary Figure S2).

For the GO terms of CC, DEGs were primarily enriched in focal adhesion, extracellular exosome, cytoplasm, cytosol, adherens junction, stress fiber, plasma membrane, cortical actin cytoskeleton, cytoskeleton, Z disc, actin filament, actin cytoskeleton, nucleus, cell junction, cortical cytoskeleton, apicolateral plasma membrane, ruffle and so on (Supplementary Figure S2).

In the MF category, DEGs were found to be enriched for protein binding, identical protein binding, cadherin binding, macromolecular complex binding, actin filament binding, protein tyrosine kinase binding, protein domain specific binding, cell adhesion molecule binding, actin binding, RNA polymerase II transcription regulatory region sequence-specific binding, transcriptional activator activity, structural constituent of cytoskeleton, GDP binding, structural constituent of muscle, protein homodimerization activity, cadherin binding involved in cell–cell adhesion, and structural molecule activity etc. (Supplementary Figure S3).

In the KEGG pathway enrichment analysis, the upregulated DEGs were enriched in tight junction, Shigellosis, Leukocyte transendothelial migration, Pathogenic *Escherichia coli* infection, Regulation of actin cytoskeleton, Fluid shear stress and atherosclerosis, Salmonella infection, Adherens junction, Mitophagy-animal, Focal adhesion, Proteoglycans in cancer, Bacterial invasion of epithelial cells, Ferroptosis, Estrogen signaling pathway, Apelin signaling pathway, Oxytocin signaling pathway, Cellular senescence, Hepatitis C, Cell adhesion molecules, Pathways in cancer, Renal cell carcinoma, Prolactin signaling pathway, Sphingolipid signaling pathway, MAPK signaling pathway, Thyroid hormone signaling pathway, etc. (Supplementary Figure S3).

### 3.3 The PPI network construction and hub genes of AP

**Figure 2:**
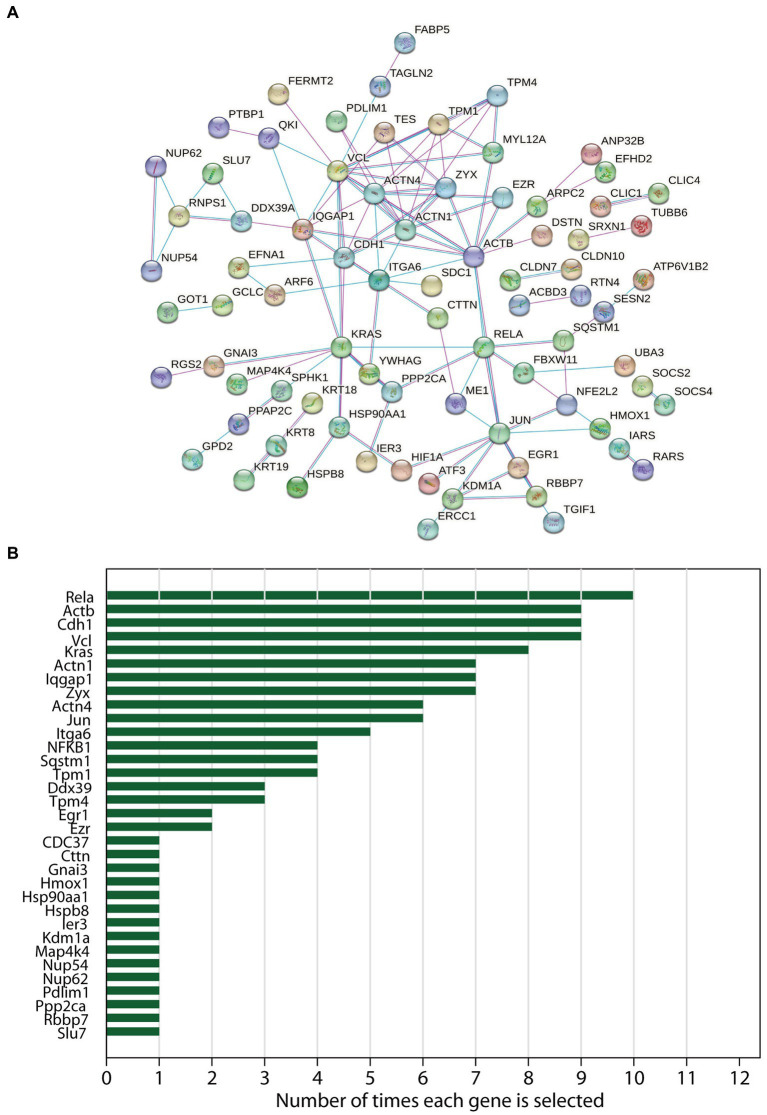
The protein–protein interaction (PPI) networks and hub genes analysis of differentially expressed genes in acute pancreatitis. **(A)** The PPI networks constructed by STRING online database. **(B)** Frequencies of occurrence of each hub gene which was selected in the 12 topological analysis methods by CytoHubba (Cytoscape plugin) were noted.

After ruling out 40 DEGs, a total of 80 DEGs were imported into the PPI network complex, which included 80 nodes and 118 edges, including upregulated and down-regulated genes (Figure 2). The results of 12 topological analysis methods are shown in Supplementary Figure S4. Among the 12 methods, the newly proposed MCC method had a better performance on the precision of predicting essential proteins from the yeast PPI network (16). The top-ranked ten genes identified by MCC were Actn4, Vcl, Actn1, Zyx, Tpm1, Actb, Cdh1, Tpm4, Rela, NF-κB (Supplementary Figure S4). Of which, Rela (10/12, 83%) was the most selected hub differentially expressed gene using all 12 topological analysis methods, followed by Actb (9/12, 75%), Cdh1 (9/12, 75%) and Vcl (9/12, 75%) (Figure 2).

### 3.4 Predictions of the potential mechanism and correlation analysis among Fabp5, TLR4 and Rela

**Figure 3:**
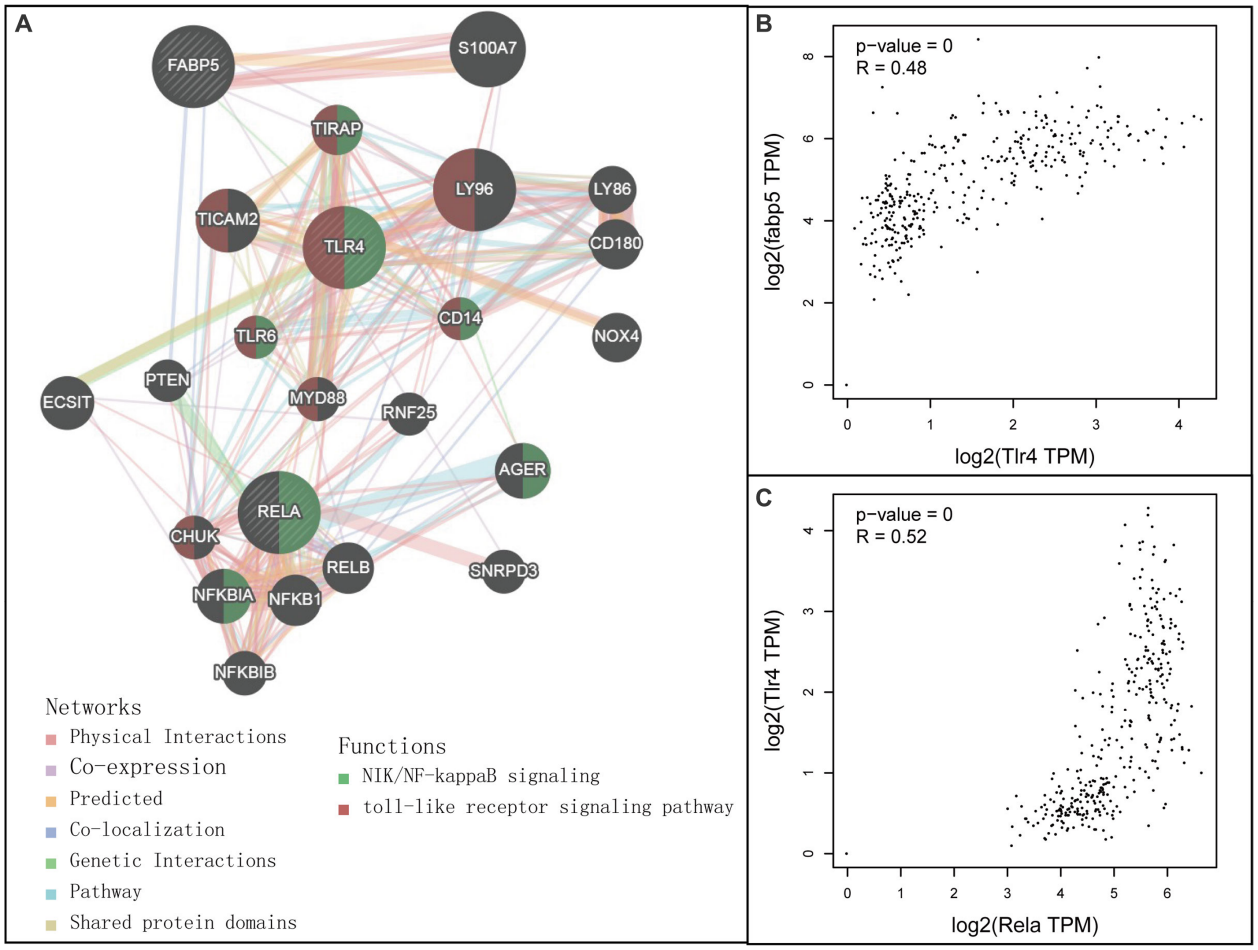
Relationship among Fabp5, TLR4 and Rela. **(A)** Interaction networks among Fabp5, TLR4 and Rela constructed by GeneMANIA (http://genemania.org). **(B)** Pearson correlation analysis between Fabp5 and TLR4 in individuals with healthy pancreaus and pancreatic adenocarcinoma. **(C)** Pearson correlation analysis between Fabp5 and Rela in individuals with healthy pancreaus and pancreatic adenocarcinoma correlation analysis.

A combined analysis of Fabp5, TLR4, and Rela through the GeneMANIA database was carried out to predict the potential interaction mechanism of these genes (Figure 3). As expected, there were genetic interactions (77.6% physical interactions) among Fabp5, TLR4, and Rela. Rela was involved in the NF-κB signaling pathway, while TLR4 was involved in both TLR signaling pathway and the NF-κB signaling pathway. Further genetic correlation analysis indicated a moderate association between Fabp5 and TLR4 (*R* = 0.48, *p* < 0.0001), as well as TLR4 and Rela (*R* = 0.52, *p* < 0.0001) (Figure 3). These results indicated that Fabp5 may regulate the severity of AP by TLR signaling and NF-κB activation.

### 3.5 Validation in different healthy human tissues and pancreatic adenocarcinoma

Expressions of Fabp5, TLR4, Rela, Actb, Cdh1, and Vcl genes in different healthy human tissues are shown in Supplementary Figure S5. The median expression levels of Fabp5 and TLR4 in the pancreas were 9.767 and 0.974 transcripts per million (TPMs), respectively. The median expression levels of Rela, Actb, Cdh1, and Vcl in the pancreas were 22.96, 404.8, 53.47, and 11.79 TPMs, respectively. Kaplan–Meier survival analysis indicated that high Rela (Logrank *p* = 0.046), Actb (Logrank *p* = 0.038), Cdh1 (Logrank *p* = 0.041) and Vcl (Logrank *p* = 0.011) gene expression was associated with poor prognosis in patients with pancreatic adenocarcinoma (Supplementary Figure S6).

### 3.6 Validation and prediction in peripheral blood in patients with AP

**Figure 4:**
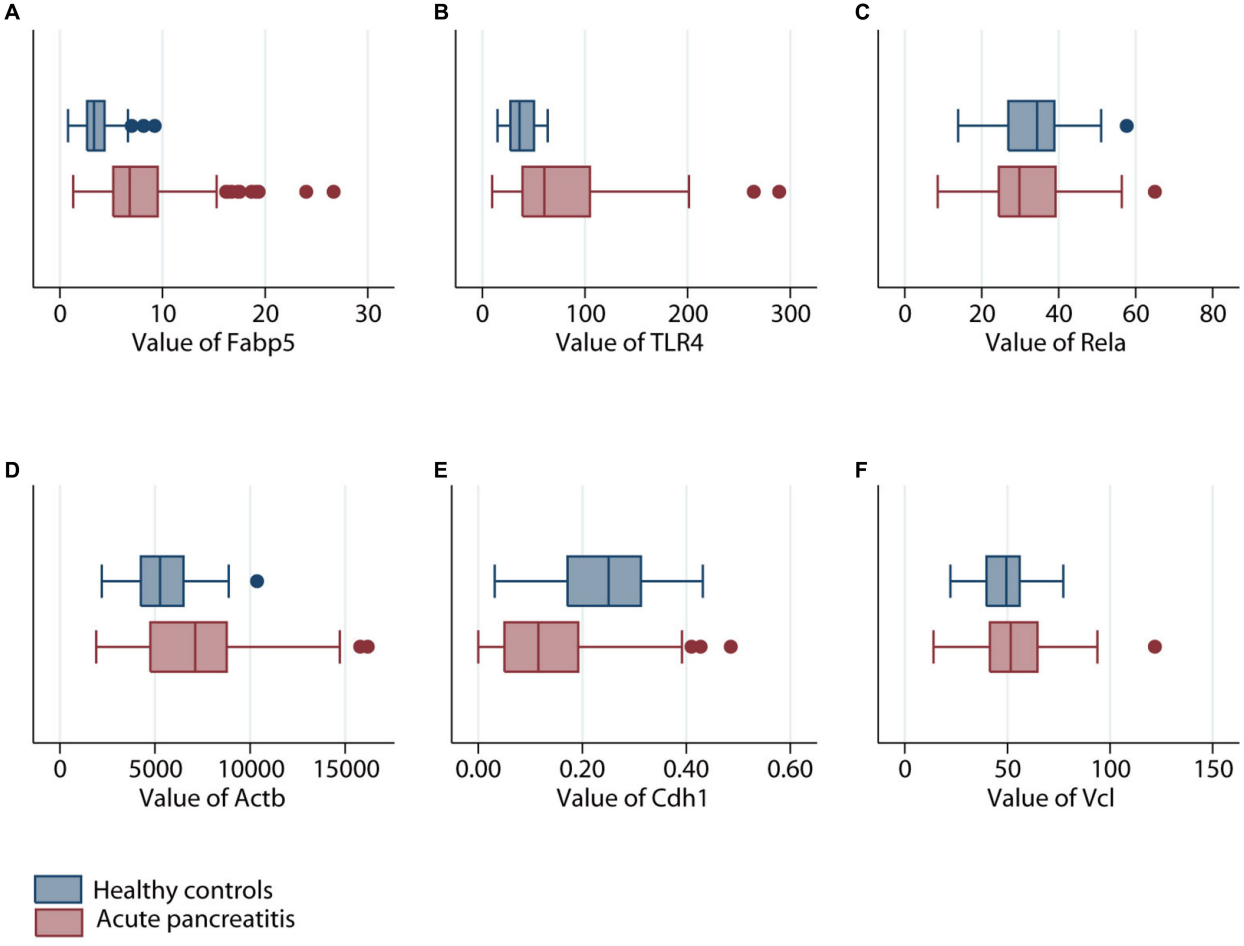
Comparison of different genes expression levels in patients with (maroon color) or without (navy color) acute pancreatitis. **(A)** Fabp5 gene. **(B)** TLR4 gene. **(C)** Rela gene. **(D)** Actb gene. **(E)** Cdh1 gene. **(F)** Vcl gene.

**Figure 5:**
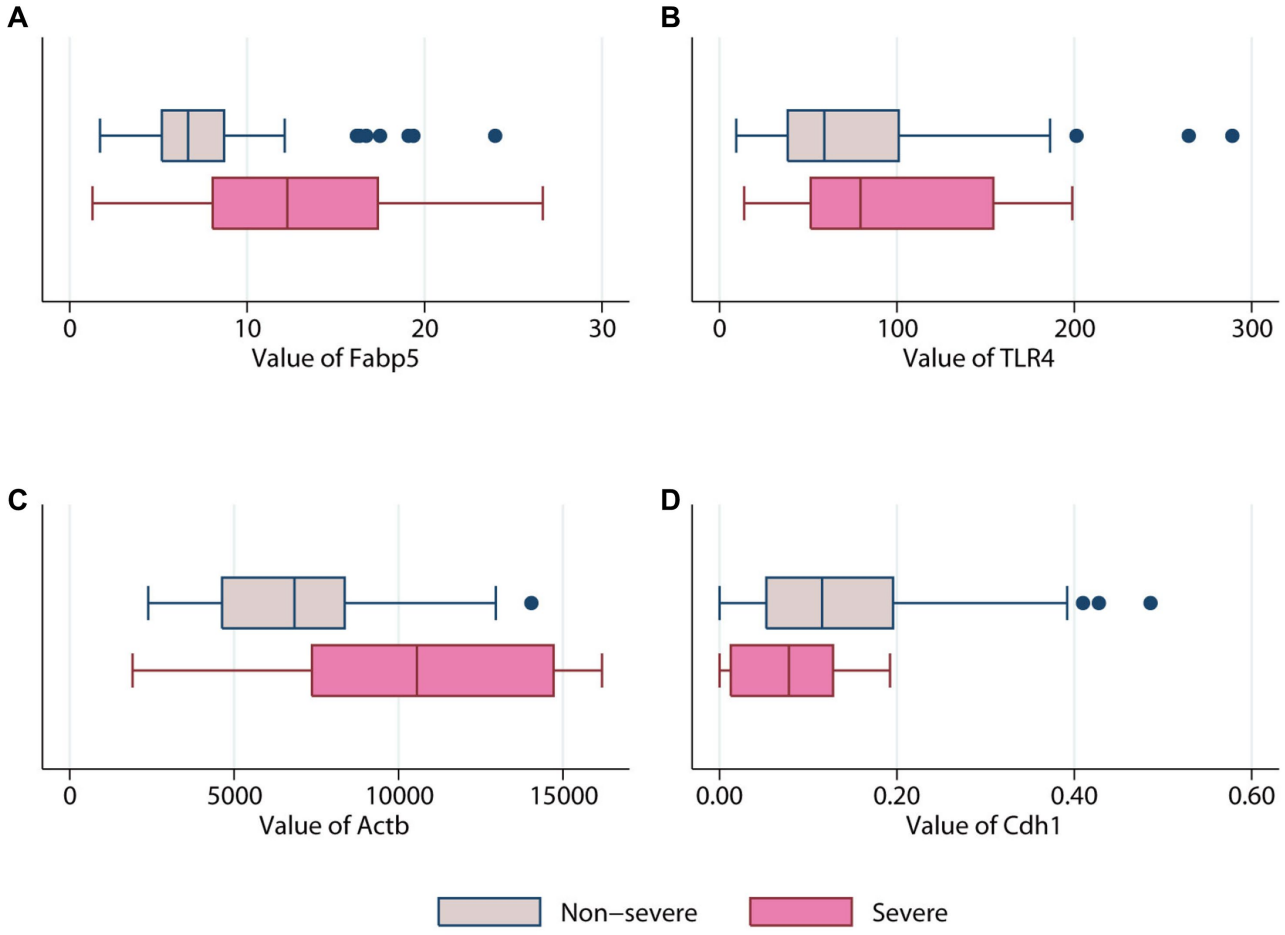
Comparison of different genes expression levels in patients with (pink color) or without (erose color) severe acute pancreatitis. **(A)** Fabp5 gene. **(B)** TLR4 gene. **(C)** Actb gene. **(D)** Cdh1.

Gene expression of Fabp5, TLR4, Rela, Actb, Cdh1, and Vcl was compared between 87 AP patients and 32 healthy volunteers (Figure 4). Gene expression of Fabp5, TLR4, and Actb was increased in AP patients when compared to healthy volunteers. Gene expression of Cdh1 was decreased in patients with acute pancreatitis when compared to healthy volunteers. There were no statistically significant differences between AP patients and healthy volunteers for Rela and Vcl gene expression (Figure 4). Further analysis indicated that patients with SAP had higher gene expression of Fabp5 and Actb than those with non-severe (namely mild or moderate–severe) AP (Figure 5).

**Figure 6:**
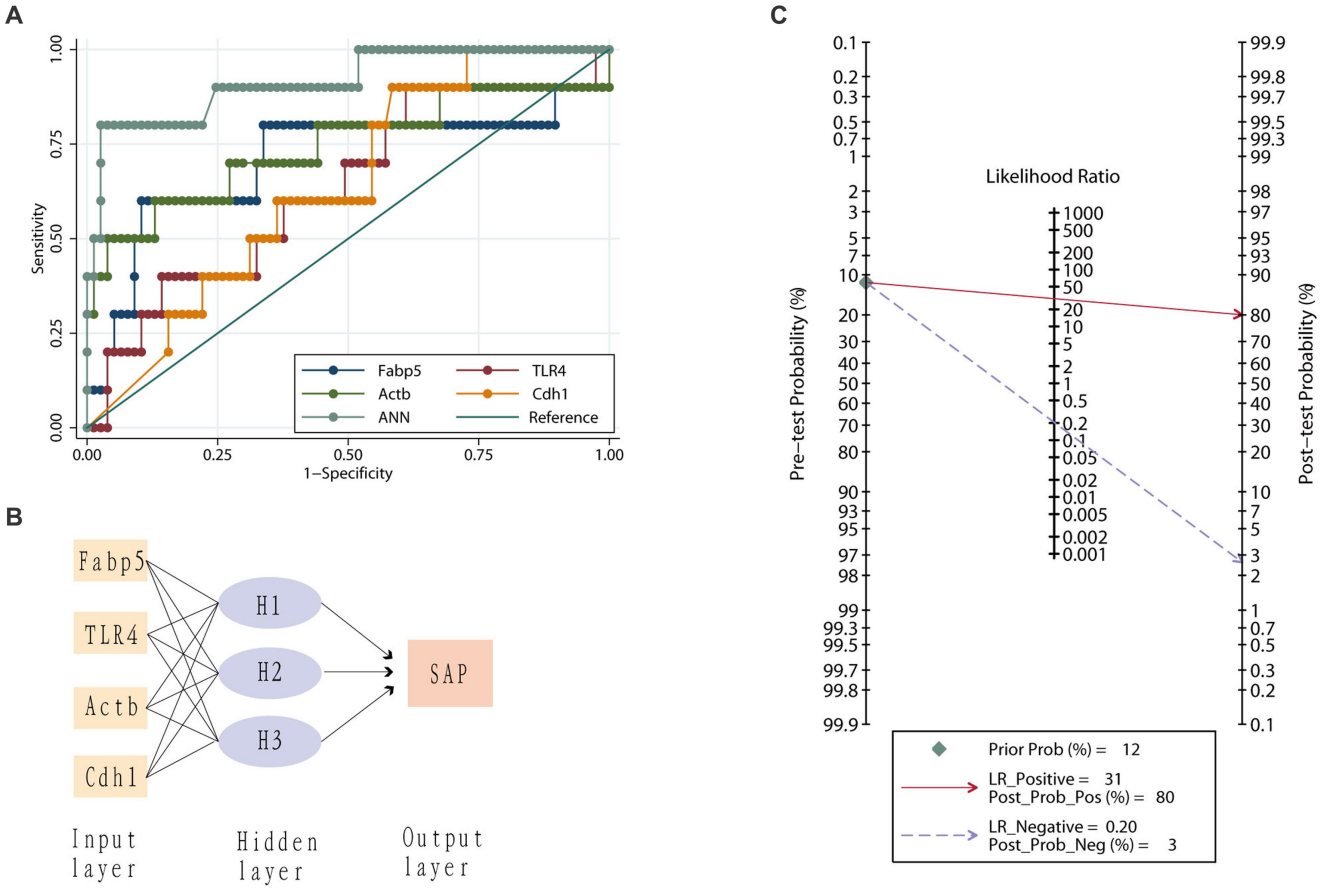
Value of different genes in predicting severe acute pancreatitis. **(A)** Receiver operating characteristic curves for the artificial neural network (ANN) model, Fabp5, TLR4, Actb and Cdh1 genes. **(B)** An artificial neural network for the prediction of severe acute pancreatitis consisting of three input variables, a hidden layer with three nodes and one output variable. **(C)** Fagan plot of artificial neural network (ANN) model for prediction of severe acute pancreatitis. Prob, probability; LR Positive, positive likelihood ratio; LR Negative, negative likelihood ratio.

The AUC for Fabp5, TLR4, Actb, and Cdh1 genes to predict SAP was 0.71 ± 0.12, 0.63 ± 0.10, 0.74 ± 0.11 and 0.64 ± 0.08, respectively (Figure 6). Therefore, both Fabp5 and Actb were useful predictors of SAP, with an AUC of more than 0.7. With a cutoff of 12.05, the sensitivity, specificity and diagnostic accuracy of Fabp5 for SAP were 60%, 89.6%, and 86.21%, respectively. With a cutoff of 9,191, the sensitivity, specificity and diagnostic accuracy of Actb for SAP were 60%, 87%, and 83.9%, respectively. A three-layer 4-3-1-feed-forward back propagation ANN model with Fabp5, TLR4, Actb and Cdh1 variables was developed and trained in 87 patients (Figure 6). The sensitivity, specificity, diagnostic accuracy, and AUC of the ANN for SAP were 80.0%, 97.4%, 95.4% and 0.91%, respectively (Figure 6). Using the SAP prevalence (11.5% in this study) as the pretest probability, the Fagan plot (Figure 6) demonstrates the clinical significance of the ANN. It provides information about the probability of being classified as organ failure, raising it to 80% when positive, and lowering the probability of SAP to 3% when negative.

### 3.7 Validation in pancreatic tissues of experimental animal model of hyperlipidemic AP

**Figure 7:**
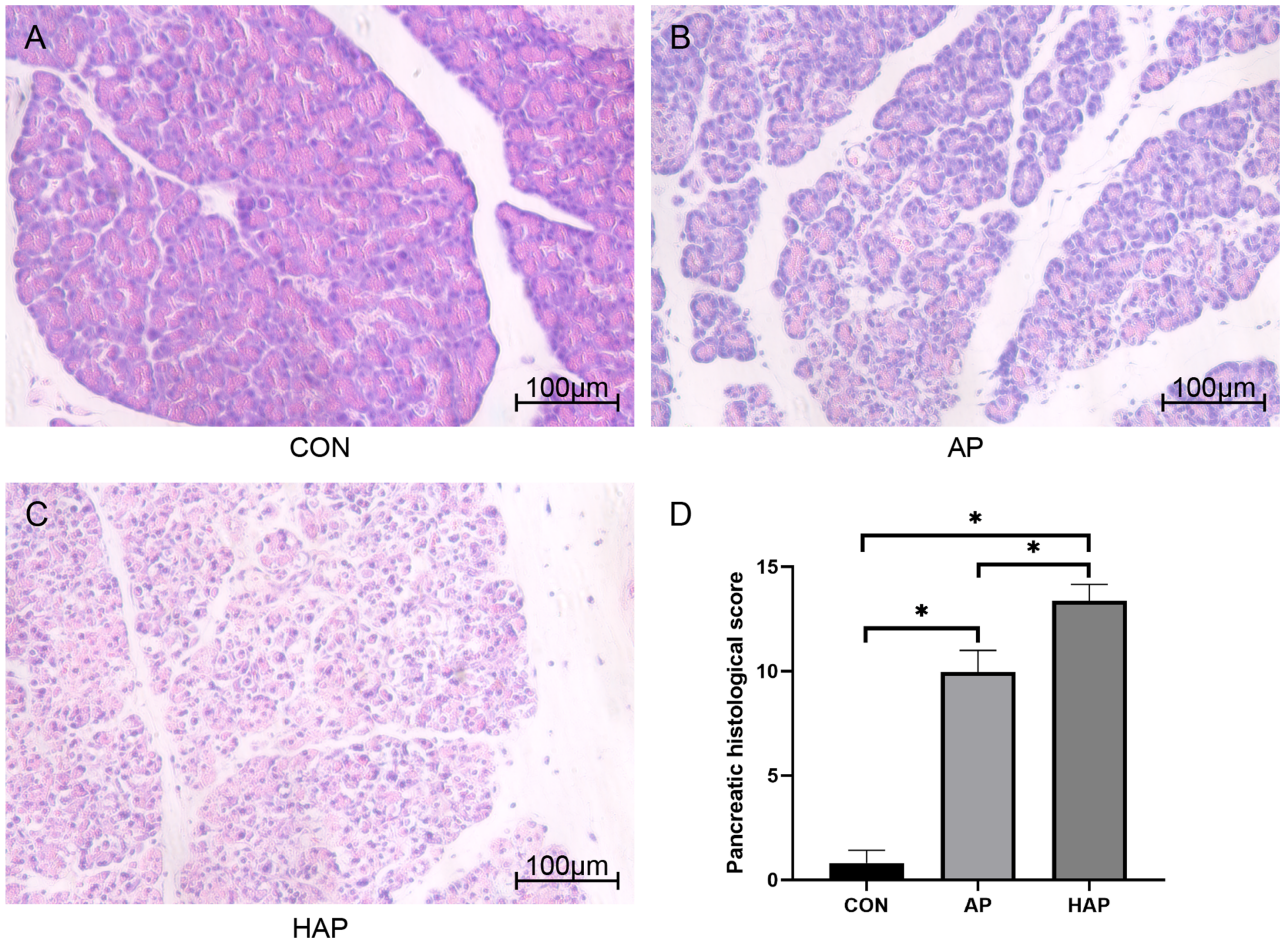
Histopathological analysis of pancreatic tissue (HE staining, ×200). **(A–C)** Edema, vacuolization, hemorrhage, inflammation, and necrosis were observed in the acute pancreatitis group (AP group) and hyperlipidemic acute pancreatitis group (HAP group) but not control group (CON group). **(D)** The statistical results of pancreatic histopathological score in different groups. *p < 0.05.

Histopathological changes of pancreatic tissues of Sprague–Dawley rats in CON group, AP group, and HAP group were investigated and are shown in Figure 7. In the AP and HAP groups, HE staining showed obvious edema, vacuolization, hemorrhage, inflammation, and necrosis in the pancreatic tissues. Pancreatic histopathological scores in the AP and HAP groups were significantly higher than those of the CON group (Figure 7, *p* < 0.05).

**Figure 8:**
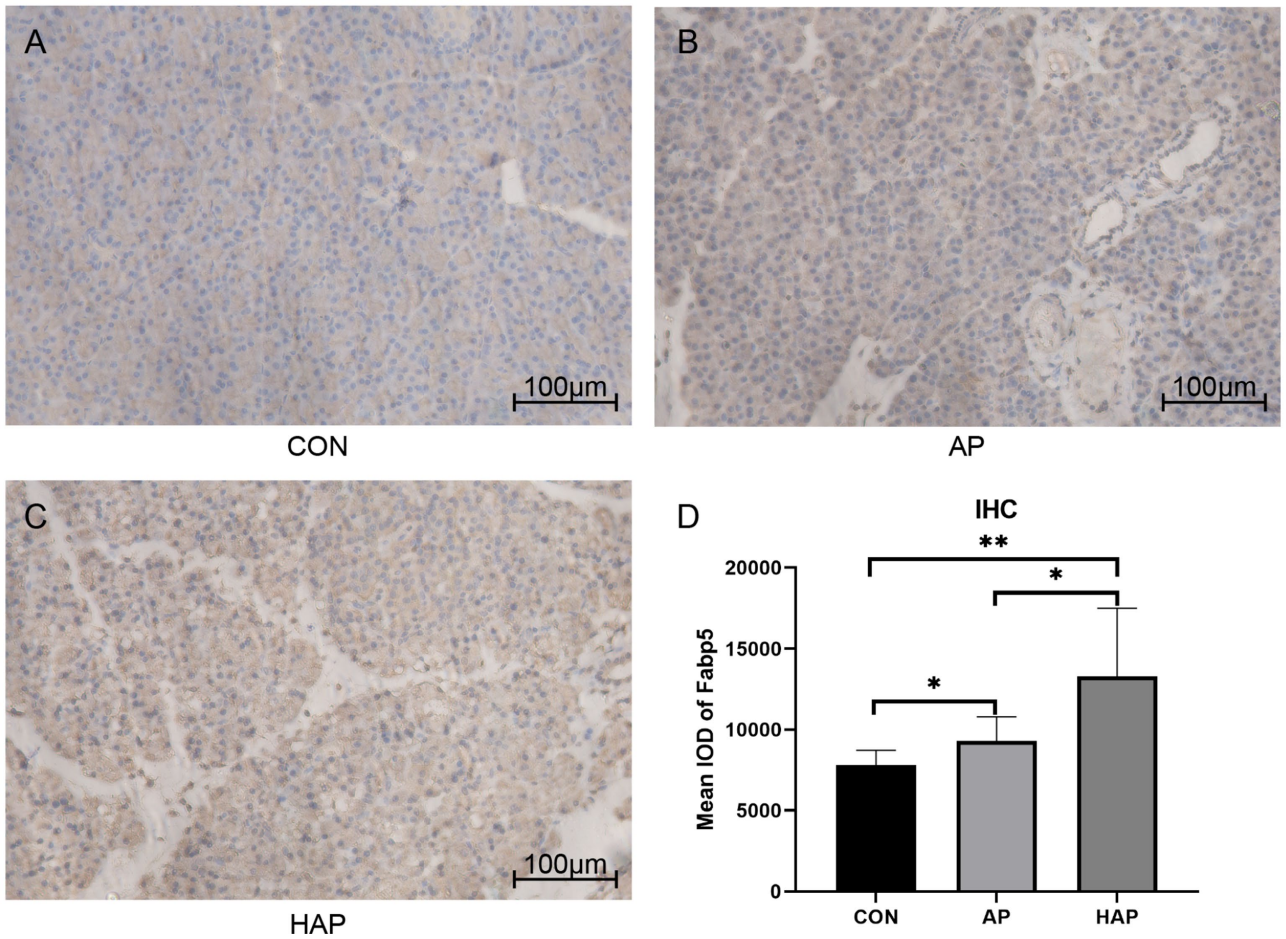
Immunohistochemistry and statistical results of the pancreatic tissue among different groups (×200). **(A–C)** The gene Fabp5 in the acute pancreatitis group (AP group) and hyperlipidemic acute pancreatitis group (HAP group) were significantly more stained than those of the control group (CON group). **(D)** Statistical analysis results of the immunohistochemical staining, which is as follows: AP group vs. CON group, *t* = 2.434, *p* < 0.05; HAP group vs. CON group, *t* = 3.588, *p* < 0.01, HAP group vs. AP group, *t* = 2.520, *p* < 0.05; *p < 0.05; **p < 0.01.

**Figure 9:**
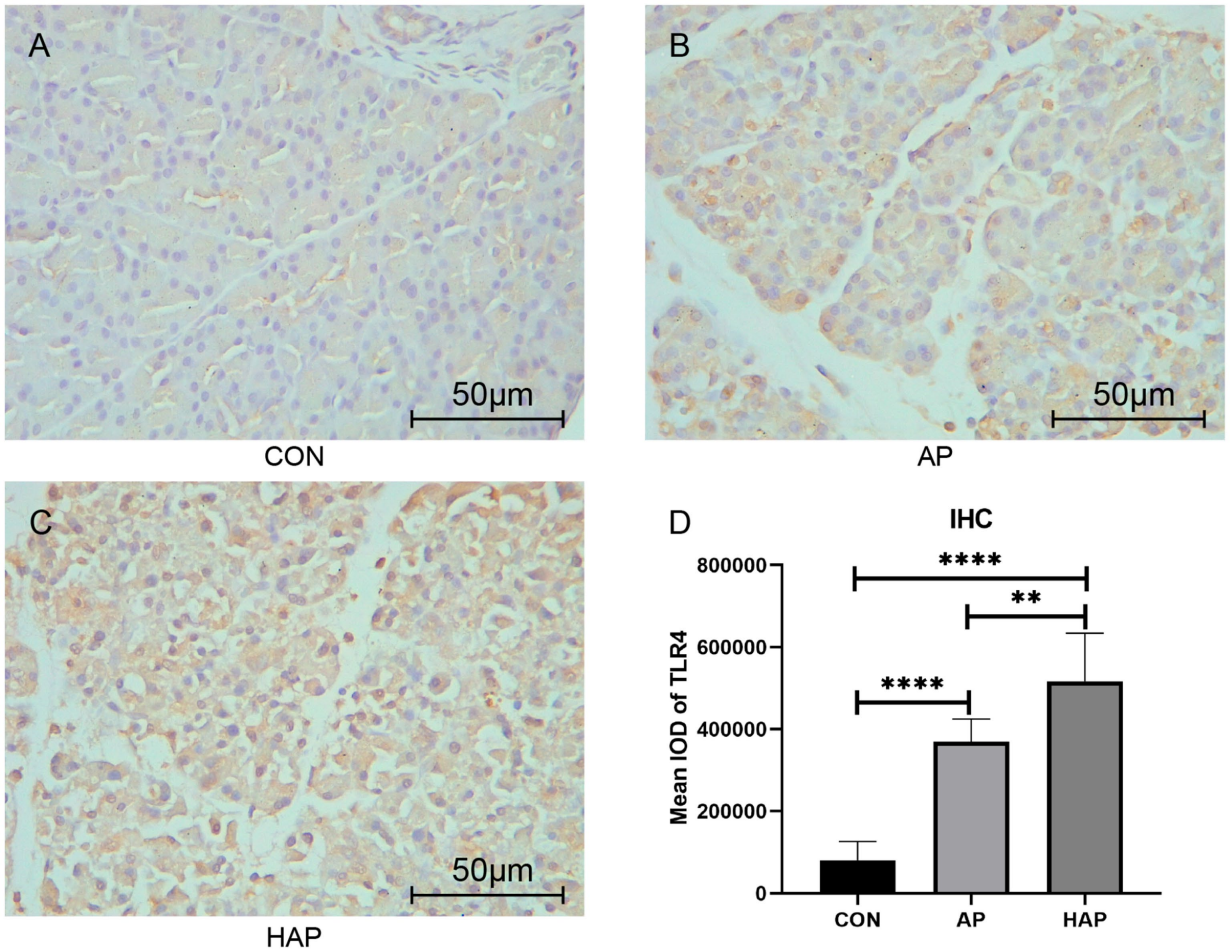
Immunohistochemistry and statistical results of the pancreatic tissue among different groups (×400). **(A–C)** The gene TLR4 in the acute pancreatitis group (AP group) and hyperlipidemic acute pancreatitis group (HAP group) were significantly more stained than those of the control group (CON group). **(D)** Statistical analysis results of the immunohistochemical staining, which is as follows: AP group vs. CON group, *t* = 12.80, *p* < 0.0001; HAP group vs. CON group, *t* = 10.96, *p* < 0.0001; HAP group vs. AP group, *t* = 3.595, *p* < 0.01; **p < 0.01; ****p < 0.0001.

Microscopic images showed that the Fabp5 and TLR4 in the AP group were significantly more stained than those in the CON group by immunohistochemical staining (Figures 8, 9). Similarly, the staining degrees of genes Fabp5 and TLR4 in the AP and HAP groups were significantly higher than those in the CON group (Figures 8, 9). In addition, the statistical analysis showed significant differences among the CON, AP, and HAP groups (Figure 8, Fabp5, AP group vs. CON group, *t* = 2.434, *p* < 0.05; HAP group vs. CON group, *t* = 3.588, *p* < 0.01, HAP group vs. AP group, *t* = 2.520, *p* < 0.05; Figure 9, TLR4, AP group vs. CON group, *t* = 12.80, *p* < 0.0001; HAP group vs. CON group, *t* = 10.96, *p* < 0.0001; HAP group vs. AP group, *t* = 3.595, *p* < 0.01).

## 4 Discussion

### 4.1 Ontology and KEGG pathway analysis in the AP

Current study suggested that DEGs enriched GO terms in Biological Process (BP) mainly covered the positive regulation of macroautophagy, positive regulation of cell migration, response to oxidative stress, transforming growth factor beta (TGFβ) receptor signaling pathway, endoplasmic reticulum unfolded protein response and tumor necrosis factor (TNF)-mediated signaling pathway (Supplementary Figure S2). It is known that the pathogenesis of AP is driven by impaired cytoprotective mechanisms (e.g., autophagy and the endoplasmic reticulum unfolded protein response). Macroautophagy recycles aged, defective or damaged cytoplasmic contents (26). Selective macro-autophagy refers to the processing and recycling of specific damaged organelles and misfolded proteins. Owing to the high efficiency in producing proteins, the mechanism of unimpaired autophagy is critical to the survival of acinar cells (27). Further study on the phenotypic changes of macrophages in the local migration has confirmed that macrophages undergo a polarization shift with M1-type macrophages (28). M1-type macrophages express high levels of proinflammatory receptors such as Toll-like receptors (TLR) and TNF receptors. They also exhibit strong activation of NF-κB transcription factors necessary for expressing proinflammatory cytokines (29, 30). The disruption of normal autophagy function in AP leads to endoplasmic reticulum stress, dysregulation of lipid metabolism, and increase in SAP (31). During endoplasmic reticulum stress, acinar cells activate the unfolded protein response to restore cellular homeostasis. This response alleviates endoplasmic reticulum stress by upregulating the endoplasmic reticulum degradation machinery for unwanted proteins, enhancing its capacity and efficiency in protein synthesis and folding (32, 33). Furthermore, injured acinar cells release chemokines, cytokines, and various adhesion molecules that recruit and regulate immune cell migration into the site of injury (34). The proinflammatory phase in AP is followed by a compensatory anti-inflammatory response syndrome, characterized by a predominance of anti-inflammatory cytokines such as TGFβ (35). Additionally, oxidative stress accelerates inflammation by triggering activation and migration of inflammatory cells, exacerbating oxidative stress and creating a vicious cycle (36). Oxidative stress also contributes to a systemic inflammatory reaction, xanthine oxidase activation, as well as glutathione and thiol oxidation (37, 38). Injury caused by oxidative stress upregulates the expression of TLR4, receptor-interacting protein 3 (RIP3), NF-κB p65, and TNF-α in the pancreatic tissue. The administration of the TLR4 inhibitor is reported to reduce inflammatory injury in pancreatic tissue, along with reduced expression of inflammatory factors RIP3, NF-κB p65, and TNF-α. Malondialdehyde and lipid peroxide levels also decrease. Meanwhile, the superoxide dismutase and glutathione levels increase (39).

In our study, DEGs were mostly enriched in some components (e.g., extracellular exosome, cytoplasm, cytosol, plasma membrane, nucleus, membrane, nucleoplasm) (Supplementary Figure S2) and functions (e.g., protein binding) (Supplementary Figure S3), for the GO terms of CC and MF. These DEGs are possibly associated with the main features of secretion and signaling of pancreatic acinar cells. Premature activation of pancreatic enzymes has been regarded as the cornerstone of pancreatitis leading to pancreatic injury (40). The acinar cells have the highest protein turnover rate, resulting in the secretion of a large volume of pancreatic enzymes, which are highly potent with the capability of hydrolysis of a wide array of organic compounds (41). On the other hand, pancreatic acinar cells secrete a variety of cytokines and inflammatory mediators under conditions of stress (e.g., oxidative stress and endoplasmic reticulum stress) in response to pancreatitis-causing stimulus, and this unique inflammatory property is critical in the robust inflammatory response in pancreatitis (42, 43).

In the KEGG pathway enrichment analysis, the upregulated DEGs were high in Shigellosis, Leukocyte transendothelial migration, Pathogenic *Escherichia coli* infection, Salmonella infection, Bacterial invasion of epithelial cells, Ferroptosis and MAPK signaling pathway (Supplementary Figure S3). Chemokines and cytokines released by injured acinar cells in pancreatitis mediate the transendothelial migration of leukocyte cells into the site of injury (34). Among these, MCP-1, the chemokine of recruiting monocytes, was upregulated by some stimulation (e.g., oleic or linoleic acid) by activating the mitogen-activated protein kinase (MAPK)/Janus kinase (JAK)-mediated NF-κB in acinar cells (44). Moreover, the gut microbiota is also strongly associated with AP (45). After the AP onsets, intestinal permeability is increased due to mucosal ischemia and the gut barrier disruption. Then gut bacteria (e.g., *Shigella* sp., pathogenic *Escherichia coli*, and Salmonella) could cross the impaired epithelial cells to reach the pancreas and systemic circulation, contributing to mortality risk in AP (46, 47). Ferroptosis has been demonstrated to contribute to SAP-induced intestinal barrier injury via lipid peroxidation-mediated intestinal epithelial cell death. Intestinal barrier damage associated with SAP as well as bacterial translocation can be ameliorated by inhibiting ferroptosis (48), suggesting that ferroptosis inhibition may be a potential therapeutic method for intestinal barrier injury in AP.

### 4.2 The roles of hub genes in AP

Rela is a significant member of NF-κB. NF-κB is a family of TF with the main function of regulating cell proliferation and apoptosis. It also plays a role in cancer and inflammation (49, 50). Previous studies have revealed that Rela plays a crucial role in the inflammatory process by promoting the release of inflammatory cytokines (51). As shown in Figure 2, Rela was considered the hub gene of AP. NF-κb is essential to the pathogenesis of AP, which is activated early in leukocytes and within pancreatic cells (37). Then actiated NF-κb releases cytokines including TNF-α and IL-6, leading to the infiltration of neutrophils and lymphocytes in the pancreas and finally leads to a persistent inflammatory response (52). Moreover, as shown in Supplementary Figure S6, survival analysis indicated that high expression of Rela was correlated with poor prognosis in pancreatic carcinoma. EMT (epithelial-to-mesenchymal transition) is a mechanism of invasion of cancer cells. NF-κb plays a role in regulating the EMT and the activation of NF-κb increased the metastasis of cancer cells via EMT induction (53).

Actb (β-actin), a member of the actin family, plays a role in cell motility, division, and cell–cell interactions (54). In this study, Figure 2 indicated that Actb was one of the hub genes of AP. Fan et al. also reported that Actb is as a member of AP hub genes (5). In addition, box plots showed that the blood gene expression of Actb was higher in patients with AP than in healthy volunteers (Figure 4). This may due to the immune infiltration of Actb. Moreover, Actb is associated with the progression of human cancers. A previous study demonstrated that Actb suppressed the growth of cells in epithelial tumors (55). Tang et al. reported that the overexpression of Actb was related to the poor overall survival duration of patients with hepatocellular carcinoma and lung adenocarcinoma (55). Moreover, as shown in Supplementary Figure S6, survival analysis indicated that high expression of Actb was associated with poor prognosis in pancreatic carcinoma. The possible explanation was that Actb may regulate cell proliferation and metastasis in cancer.

Cdh1 (E-cadherin) belongs to the cadherin family, a superfamily of adhesion molecules, which plays a significant role in a range of functions, including mechanical adhesion between cells, tissue morphogenesis and tissue homeostasis (56). In our study, Cdh1 was considered a member of hub genes of AP (Figure 2), which was consistent with the results from Zhong et al. and Ji et al. (6, 7). As shown in Figure 4, Cdh1 gene expression was decreased in AP patients when compared to healthy volunteers. This may be due to impaired cell–cell adhesion of Cdh1. A mouse model produced by Kaneta et al. also showed that loss of Cdh1 led to the formation of ducts with apoptotic changes and resulted in pancreatitis-like changes (57). Previous studies showed the role of Cdh1 in different types of cancers. For example, Elangovan et al. demonstrated that Cdh1 suppresses IGF1R signaling in lobular breast carcinoma (58). Survival analysis indicated that high expression of Cdh1 was associated with poor prognosis in patients with pancreatic adenocarcinoma (Supplementary Figure S6).

Vcl (Vinculin) plays a significant role in cell–cell and cell-matrix adhesion and cell migration (59). In our study, Vcl was considered one of the hub genes of AP (Figure 2), which was consistent with the result reported by Fan et al. (5). In addition, Vcl is associated with the development of cancers. Wang et al. demonstrated that high expression of Vcl was related to the prognosis of patients with gastric cancer. Vcl reduced the expression of EPCAM (an adhesion molecule of epithelial cells), and promoted cell migration (60). Zhou et al. showed that Vcl was correlated with unfavorable prognosis (61). As shown in Supplementary Figure S6, survival analysis indicated that high expression of Vcl was associated with poor prognosis in pancreatic carcinoma. Similarly, Shi et al. indicated that overexpression of Vcl eliminated the Linc01060-mediated repression of the cell proliferation and invasion in pancreatic cancer (62).

### 4.3 The possible effects of Fabp5 in high-cholesterol diet associated AP

Fabp5 is a cytoplasmic protein mainly involved in the uptake, transport, and metabolism of fatty acids in the cytoplasm. The current study suggested that “Fabp5” gene was upregulated in AP but down-regulated in high-cholesterol diet compared with the control (Figure 1). The level of Fabp5 gene was positively correlated with the severity of AP (Figures 4, 5). Moreover, the immunohistochemical staining suggested that Fabp5 expression was markedly increased in pancreatic tissues of the experimental pancreatitis model (Figures 7, 8). In the study of Wu et al. (63), the production of the Fabp5 protein in a human retinal pigment epithelium (RPE) cell line was inhibited by using RNA interference technology. As a result, cholesterol and cholesterol ester were decreased by about 40%, whereas free fatty acids and triglycerides were increased by 18% and 67% after siRNA treatment, respectively. One possible explanation for the decreased cellular levels of cholesterol and cholesterol esters is that fatty acids are critical in a variety of cellular processes, including mitochondrial β-oxidation (64). Fabps are thought to transport fatty acids to the mitochondria for β-oxidation (65). Owing to the disruption of intracellular transport caused by Fabp5 mRNA knockdown, fatty acids may not be transported to the mitochondria for β-oxidation. Under these circumstances, the levels of acetyl coenzyme A (CoA) derived from β-oxidation for cholesterol synthesis would be expected to decrease, resulting in decreased cholesterol levels. This is because cholesterol is primarily synthesized from acetyl CoA through the 3-hydroxy-3-methyl glutaryl (HMG)-CoA reductase pathway (66). Therefore, high-fat diet increases cholesterol in peripheral blood, and in order to maintain cholesterol levels, the endogenous synthesis of cholesterol may be reduced by inhibiting Fabp5 mRNA expression. On the other hand, in patients with high cholesterol diet associated with AP, it was speculated that the body undergoes a state of systemic inflammatory response, which may result in increasing fat mobilization and hence, altered Fabp5 expression in peripheral blood.

Current study indicated genetic interactions between Fabp5, TLR4 and Rela. Fabp5 may regulate the severity of AP through TLR signaling modulation and via the Nuclear Factor-κB pathway (Figure 3). The immunohistochemical staining suggested that TLR4 expression was markedly increased in pancreatic tissues of the experimental animal model (Figure 9). Hou et al. (67) found that myeloid-specific Fabp5 knockout alleviated lipopolysaccharide (LPS)-induced acute liver injury and reduced levels of proinflammatory cytokines, including TNF-α, IL-6, and IL-1β by activating AMP-activated protein kinase (AMPK), and inhibited the NF-κB pathway during the inflammatory response of macrophages. Senga et al. (68) suggested that FABP5 promotes lipolysis of lipid droplets, *de novo* fatty acid synthesis and activation of NF-kappaB signaling in cancer cells. TLR4 recognizes a series of exogenous and endogenous ligands, transduces extracellular signals into the cell, and thus mediates inflammation (69). Besides, TLR4 is widely expressed in pancreatic tissues, and TLR4 deficiency reduces acinar cell necrosis and attenuates the AP severity (11, 70). TLR4 could also regulate chemokine formation, neutrophil recruitment, and tissue damage in mice with SAP (71). Previous reports indicate that free fatty acids, especially saturated fatty acids, can activate TLR4-mediated proinflammatory signaling pathways (72, 73). Hong et al. reported that increasing serum free fatty acids levels was associated with upregulation of TLR4 and activation of NF-κB in high-fat diet-associated AP (39). The activation of nuclear factor-kappaB in acinar cells has been shown to increase SAP in mice (74). Inhibition of TLR4 signal transduction exerts protective effects *in vitro,* evident from the pancreatic acinar cells of AP mice (75, 76).

### 4.4 The prediction in blood for SAP

Patients with SAP have a high mortality rate and are frequently moved to the critical care unit. As a result, it is critical to identify predictors of severe illness in the early stages of AP in order to identify individuals who might benefit the most from better surveillance or early therapies. Many clinical scoring systems for illness severity prediction have been introduced, such as the Japanese Severity score (JSS) (77), the BISAP (78), and the Pancreatitis Outcome Prediction (POP) Score (79). However, the existing scoring systems have moderate accuracy in predicting the SAP (80). Several laboratory indexes such as low-density lipoprotein cholesterol (81) and blood urea nitrogen (BUN) (82) have been proposed as single predictors of AP severity. However, these single prediction markers are easy to use in practice but lack high accuracy. To the best of our knowledge, the use of hub genes in predicting disease severity in AP patients has not been evaluated yet.

Current study showed that the AUC for Fabp5 and Actb predicting SAP were 0.71 ± 0.12 and 0.74 ± 0.11, respectively (Figure 6). This means that either Fabp5 or Actb could be used as a single predictor of SAP in practice. With a cutoff of 12.05, specificity and diagnostic accuracy of Fabp5 for SAP were 89.6%, and 86.21%, respectively. However, the sensitivity (60%) is low, which means that some of these patients who were at high risk of developing SAP may not have a high expression of Fabp5 in peripheral blood in patients with AP. An ANN consisting of Fabp5, TLR4, Actb and Cdh1 markedly improved performance in predicting SAP (Figure 6). The sensitivity, specificity, and diagnostic accuracy of the ANN for SAP were 80.0%, 97.4% and 95.4%, respectively. Using the prevalence of SAP (11.5% in this study) as the pretest probability, the Fagan plot (Figure 6) shows that ANN can be clinically informative because it increases the probability of being classified as organ failure to 80% when positive and lowers the probability to 3% when negative. This implies that a positive ANN result in patients with a high expression of Fabp5, TLR4, and Actb, and a low expression of Cdh1 in peripheral blood, indicates a higher risk of developing SAP. Such individuals would likely benefit the most from close surveillance or aggressive intervention.

### 4.5 Strengths and limitations

To the best of our knowledge, this is first study to investigate common DEGs between high-cholesterol diet and AP. In addition, Rela was first time identified as the most important hub gene of AP. At last, Fabp5 and hub genes of AP also were first time evaluated as potential predictors of SAP. Our study has several limitations. First, the sample size used to identify the predictors and construct ANN model for SAP was relatively small, introducing the possibility of bias. Moreover, the performance of our ANN model lacked validation on an external dataset. Therefore, an external prospective validation in a larger, independent patient population is imperative before considering its application in clinical practice. Secondly, the variables (Fabp5, TLR4, Actb, and Cdh1) employed in the ANN model are not routinely available in most emergency situations, potentially limiting the general applicability of the model in clinical practice. Thirdly, our study lacked data on certain clinical or experimental results such as pleural effusion, blood urea nitrogen, and commonly used scoring systems like the BISAP. Future studies should consider comparing our ANN model with such single predictors and scoring systems. Lastly, although we conducted immunohistochemical staining experiments *in vivo* to verify the role of Fabp5 in AP, the mechanisms underlying how hypercholesterolemia affects the expression of Fabp5 and how Fabp5 exacerbates the severity of AP have not been validated *in vivo* and *in vitro*. Therefore, exploring these mechanisms, including signaling pathways and relationships among Fabp5, TLR4, and Rela, requires further well-designed experiments with randomization, blinding, inter-observer reliability, and stringent adherence to ethical guidelines, such as Real-time Reverse Transcription-Polymerase Chain Reaction (RT-qPCR) studies.

## 5 Conclusion

Among the DEGs, Fabp5 was the sole gene identified as common between a high-cholesterol diet and AP. Additionally, Rela, Actb, Cdh1, and Vcl were identified as hub genes associated with AP. The impact of a high-cholesterol diet on the severity of AP appears to be mediated through the regulation of Fabp5 expression, potentially leading to increased activation of TLR signaling and the Nuclear Factor-κB pathway. Furthermore, an ANN model comprising Fabp5, TLR4, Actb, and Cdh1 in peripheral blood demonstrated utility in predicting SAP.

## Data availability statement

The original contributions presented in the study are included in the article/Supplementary materials, further inquiries can be directed to the corresponding author.

## Ethics statement

The studies involving humans were approved by the Ethics Committee of the First Affiliated Hospital of Wenzhou Medical University. The ethics committee/institutional review board waived the requirement of written informed consent for participation from the participants or the participants' legal guardians/next of kin because all the data about human research in this study come from public database. The animal study was approved by Wenzhou Medical University Animal Policy and Welfare Committee. The studies were conducted in accordance with the local legislation and institutional requirements.

## Author contributions

MQ: Writing – original draft, Investigation. FC: Software, Writing – review & editing. YH: Writing – original draft. LS: Writing – original draft. JL: Investigation, Methodology, Validation, Writing – review & editing. WW: Writing – review & editing. ZB: Writing – review & editing. MZ: Writing – review & editing. HG: Writing – review & editing. JP: Writing – review & editing. WH: Conceptualization, Data curation, Formal analysis, Funding acquisition, Investigation, Methodology, Project administration, Resources, Software, Supervision, Validation, Visualization, Writing – original draft, Writing – review & editing.

## Footnotes

^1^ https://www.ncbi.nlm.nih.gov/geo/geo2r/

^2^ http://davidd.ncifcrf.gov/

^3^ http://string-db.org

^4^ http://genemania.org

^5^ http://gepia.cancer-pku.cn/

^6^ https://www.gtexportal.org/home/
