# Supplementary material for: Fabp5 is a common gene between a high-cholesterol diet and acute pancreatitis
Source: Front Nutr. 2023 Dec 22;10:1284985. doi: 10.3389/fnut.2023.1284985 (PMC10768664; doi:10.3389/fnut.2023.1284985)
Supplement: Supplementary file 1 [file Data_Sheet_1.pdf]

Supplementary Table 1. Microarray data information of acute pancreatitis associated dataset.

	Control group	Acute pancreatitis group
**GSE3644**	GSM84549 GSM84550 GSM84551	GSM84552 GSM84553 GSM84554
**GSE65146**	GSM1588086 GSM1588087 GSM1588088 GSM1588089 GSM1588090	GSM1588094 GSM1588095 GSM1588096
**GSE109227**	GSM2935589 GSM2935590 GSM2935591 GSM2935592 GSM2935593	GSM2935594 GSM2935595 GSM2935596 GSM2935597 GSM2935598 GSM2935599
**GSE194331**	GSM5833650 GSM5833651 GSM5833652 GSM5833653 GSM5833654 GSM5833655 GSM5833656 GSM5833657 GSM5833658 GSM5833659 GSM5833660 GSM5833661 GSM5833662 GSM5833663 GSM5833664 GSM5833665 GSM5833666 GSM5833667 GSM5833668 GSM5833669 GSM5833670 GSM5833671	GSM5833563 GSM5833564 GSM5833565 GSM5833568 GSM5833569 GSM5833573 GSM5833574 GSM5833575 GSM5833576 GSM5833577 GSM5833579 GSM5833581 GSM5833583 GSM5833584 GSM5833585 GSM5833586 GSM5833588 GSM5833589 GSM5833590 GSM5833591 GSM5833593 GSM5833594 GSM5833595 GSM5833597 GSM5833599 GSM5833601 GSM5833602 GSM5833603 GSM5833604 GSM5833606 GSM5833607 GSM5833608 GSM5833609 GSM5833611 GSM5833612 GSM5833613 GSM5833615 GSM5833616 GSM5833617 GSM5833618 GSM5833619 GSM5833621 GSM5833622 GSM5833623

	GSM5833672 GSM5833673 GSM5833674 GSM5833675 GSM5833676 GSM5833677 GSM5833678 GSM5833679	GSM5833624 GSM5833625 GSM5833626 GSM5833627 GSM5833630 GSM5833631 GSM5833632 GSM5833633 GSM5833634 GSM5833645 GSM5833646 GSM5833648 GSM5833649
		GSM5833566 GSM5833567 GSM5833570 GSM5833571 GSM5833587 GSM5833592 GSM5833596 GSM5833598 GSM5833600 GSM5833605 GSM5833610 GSM5833614 GSM5833629 GSM5833635 GSM5833636 GSM5833637 GSM5833638 GSM5833640 GSM5833641 GSM5833643
		GSM5833572 GSM5833578 GSM5833580 GSM5833582 GSM5833620 GSM5833628 GSM5833639 GSM5833642 GSM5833644 GSM5833647

Datasets GSE3644, GSE65146, and GSE109227 were based on the platform GPL339 (Affymetrix Mouse Expression 430A Array). Dataset GSE194331 was gene expression profiling of acute pancreatitis. In this dataset, peripheral blood was collected from 87 patients with acute pancreatitis of varying severity (mild=57, moderate-severe=20, severe=10) within 24 hours of presentation to the hospital and from 32 healthy controls.

Supplementary Table 2. Microarray data information of high-cholesterol diet associated dataset.

	Normal Diet	High-Cholesterol Diet
GSE159656	GSM4836988	GSM4836976,
	GSM4836989	GSM4836977,
	GSM4836990	GSM4836978

Dataset was based on the platform GPL6246 (Affymetrix Mouse Gene 1.0 ST Array), which includes mouse models that were fed either with a high-cholesterol diet or normal diet as control.

Supplementary Table 3. All 141 common differentially expressed genes (DEGs) were detected from three profile datasets, including 130 upregulated genes and 11 down-regulated genes identified from the three datasets of Acute Pancreatitis.

**DEGs**	**Gene Names**
Upregulated	Gclc Trp53inp1 Prdm4 Pvr Krt8 Tagln2 Krt18 Tgif1 Ddr1 Ier3 Tubb6 Sdc1 Rcan1 Csrp1 Cdc42ep5 Dstn Egr1 Efna1 Cdh1 Pafah1b2 Sphk1 Rras2 Tjp2 Ier5 Atf3 Rtn4 Iqgap1 Sat1 Efhd2 Fbxw11 Tnfrsf12a Socs2 Grb10 Klf6 Fermt2 Actb Sesn2 Rgs2 Tmsb10 Me1 Zyx Ddx39 Itga6 Gpd2 Rars Hmox1 Clic4 Ywhag 7-Mar Puf60 Ccdc71l Rela Nabp1 Actn4 Qk Zfp52 Kras Got1 Krt19 Clic1 Tubb2a Btg2 Rbbp7 Jun Osmr Tpm4 Sft2d2 Cast Acvr1 Mat2a Arf6 Ppp2ca Vcl Slc20a1 Uba3 Klf7 Zwint Ptbp1 Epcam Iars Ceacam1 Myl12a Zfand5 Srxn1 Fabp5 Dusp16 Ptpn12 Prep Map4k4 Nedd9 Nup54 Plpp2 Kctd10 Hsp90aa1 Slc2a1 Coq8b Ezr Msmo1 Pdlim1 Cxadr Rhob Acsl4 Nup62 Atp6v1b2 Actn1 Ercc1 Nfe2l2 Socs4 Anxa3 Rnps1 Il17ra 2810474O19Rik Hif1a Kdm1a Gadd45b Plp2 Arrdc4 Appbp2 Tes Gnai3 Arpc2 Cldn7 Tpm1 Gcnt2 Anp32b Sqstm1 Slu7 Cttn Hspb8 Acbd3
Down-regulated	Upp2 Sostdc1 Klf15 Cbfa2t3 Dhrs3 Ivd Herpud1 Sel1l Dbp Cldn10 Bhlha15

Supplementary Figure 1

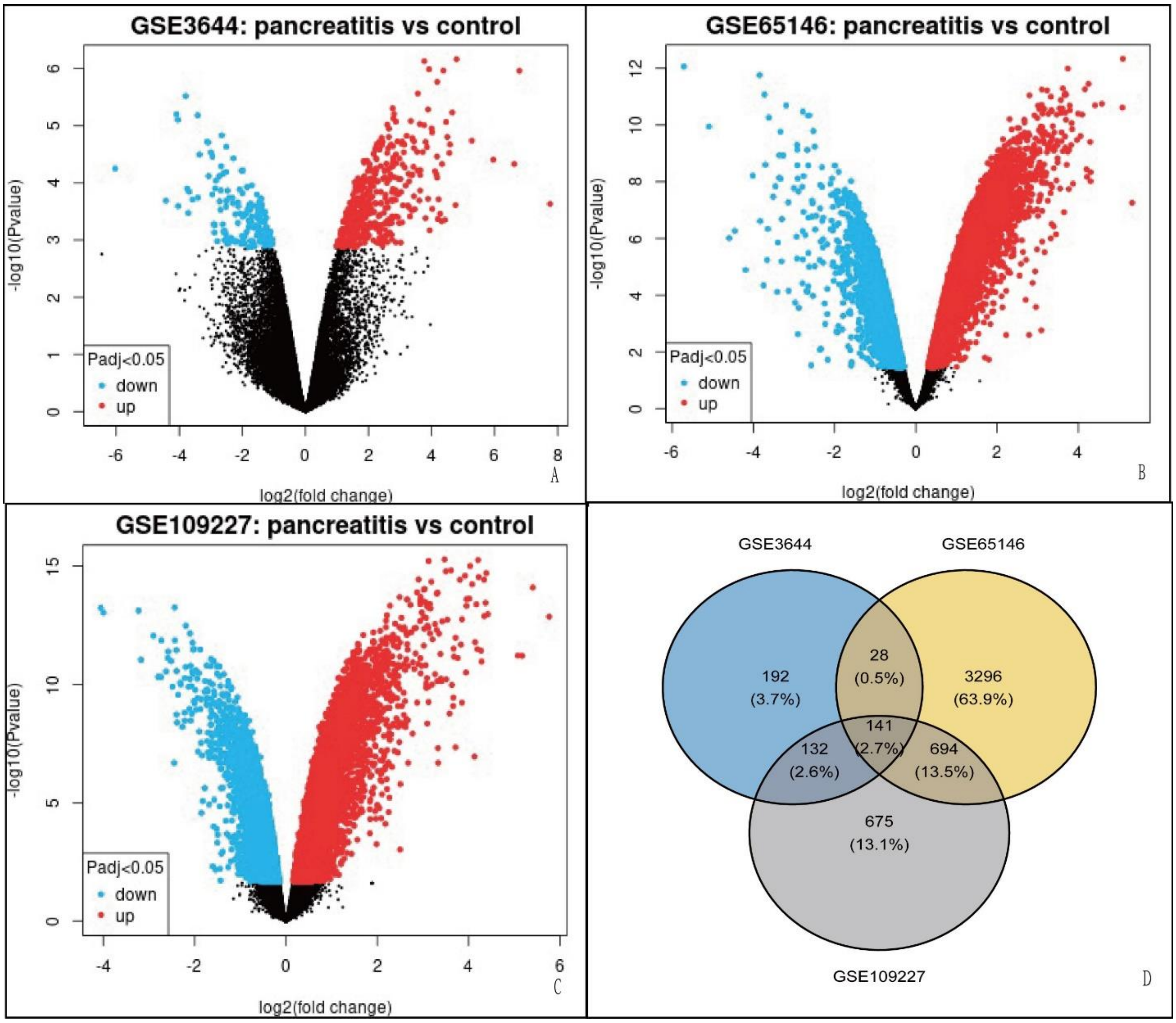

Supplementary Figure 1. Identification of common differentially expressed genes for acute pancreatitis. (A) Volcano plot of differentially expressed genes in GSE3644 microarrays. Red plot indicates genes with high levels of expression, green plot indicates genes with low levels of expression, and black indicates plot genes with no differentially of expression based on the criterion of P value < 0.05 and |log FC| > 1.0, respectively. (B) Volcano plot of differentially expressed genes in GSE65146 microarrays. Red plot indicates genes with high levels of expression, green plot indicates genes with low levels of expression, and black indicates plot genes with no differentially of expression based on the criterion of P value < 0.05 and |log FC| > 1.0, respectively. (C) Volcano plot of differentially expressed genes in GSE109227 microarrays. Red plot indicates genes with high levels of expression, green plot indicates genes with low levels of expression, and black indicates plot genes with no differentially of

expression based on the criterion of P value < 0.05 and |log FC| > 1.0, respectively. (D) The Venn diagram of overlapping genes differentially expressed in three datasets.

Supplementary Figure 2

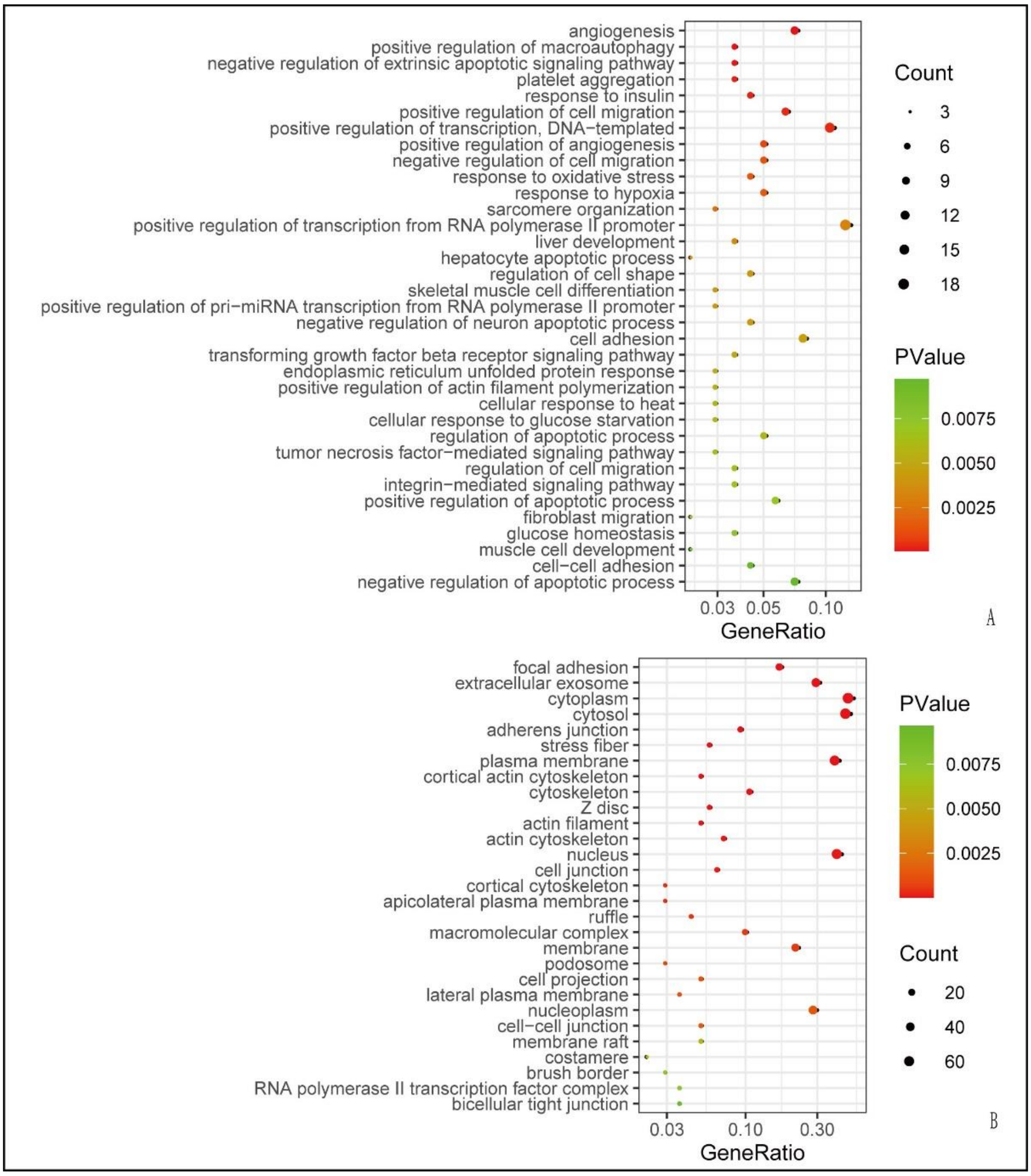

Supplementary Figure 2. Functional and pathway enrichment analysis of differentially expressed genes. (A) biological process (BP), (B) cellular component (CC). The size of the dots represents the count of the enriched differentially expressed genes, and the dot color represents the P value.

Supplementary Figure 3

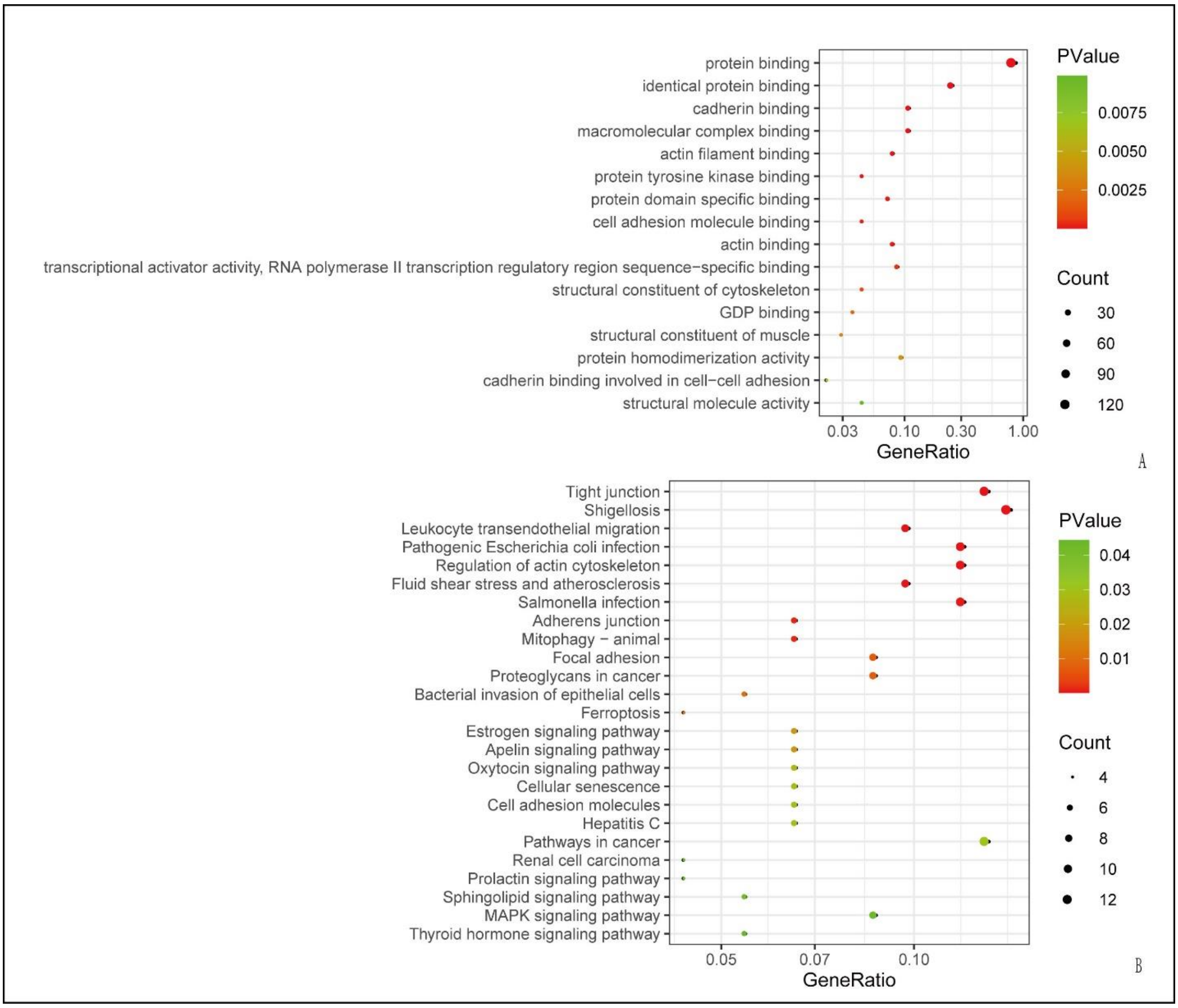

Supplementary Figure 3. Functional and pathway enrichment analysis of differentially expressed genes. (A) molecular function (MF) categories, (B) KEGG pathways. The size of the dots represents the count of the enriched differentially expressed genes, and the dot color represents the P value.

Supplementary Figure 4

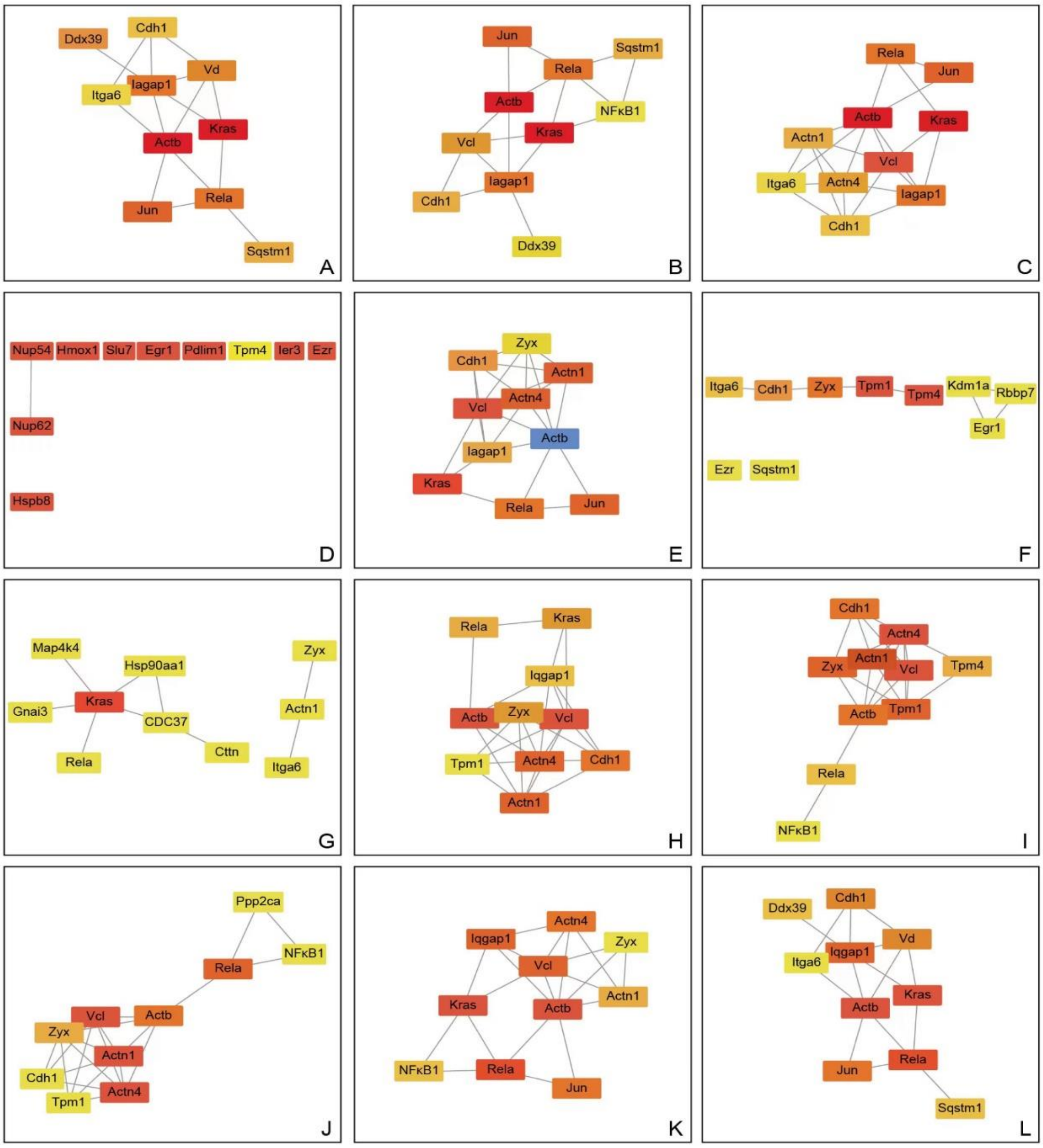

Supplementary Figure 4. Results of hub genes identified through 12 topological analysis methods by CytoHubba (Cytoscape plugin). (A) Betweenness. (B) BottleNeck. (C) Closeness. (D) ClusteringCoefficient. (E) Degree. (F) Density of Maximum Neighborhood Component. (G) EcCentricity. (H) Edge Percolated Component. (I) Maximal Clique Centrality. (J) Maximum Neighborhood Component. (K) Radiality. (L) Stress.

Supplementary Figure 5

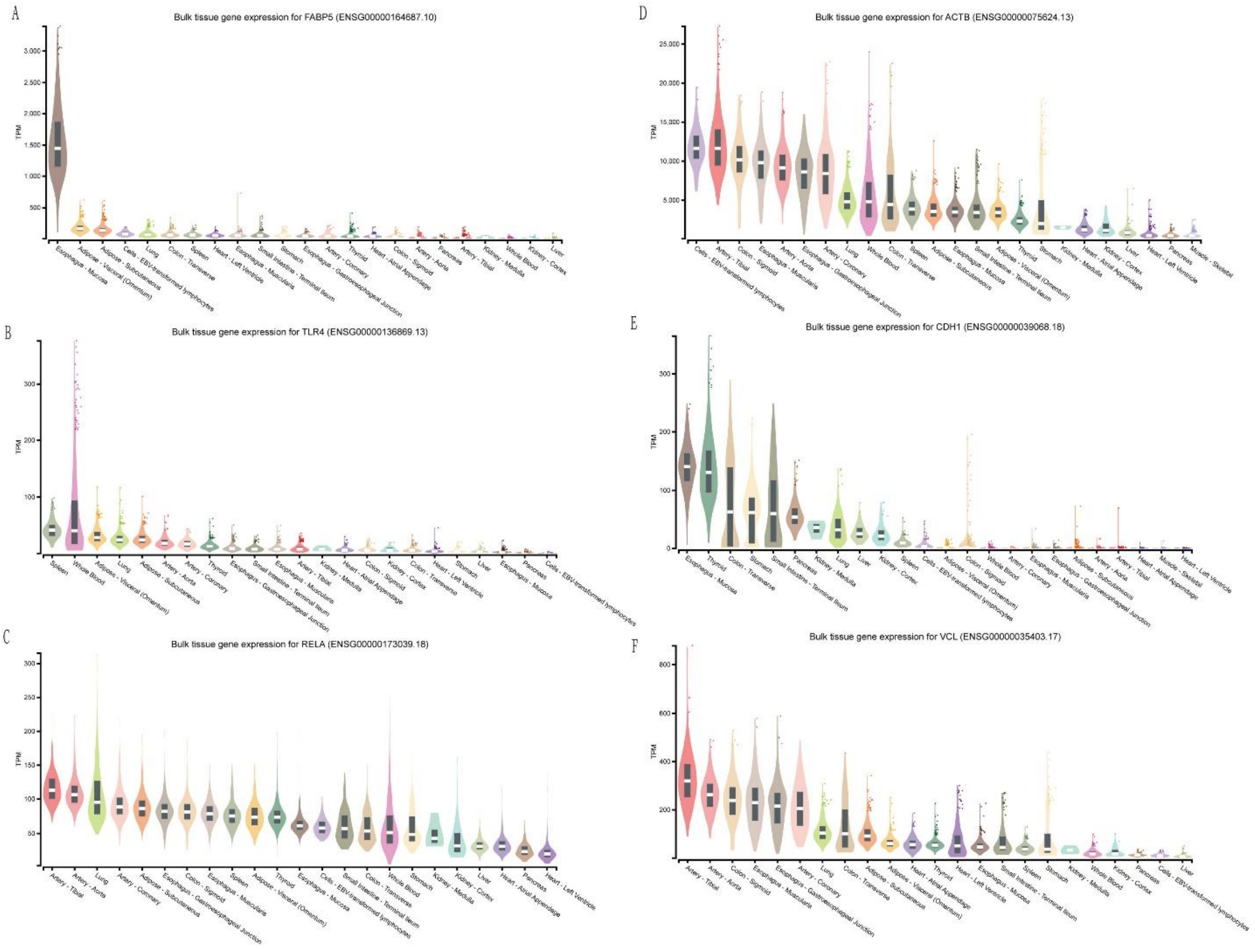

Supplementary Figure 5. Expressions of Fabp5, TLR4, Rela, Actb, Cdh1 and Vcl genes in different healthy human tissues, which were evaluated with genotype-tissue expression (GTEx) project dataset from The Human Protein Atlas database.

Supplementary Figure 6

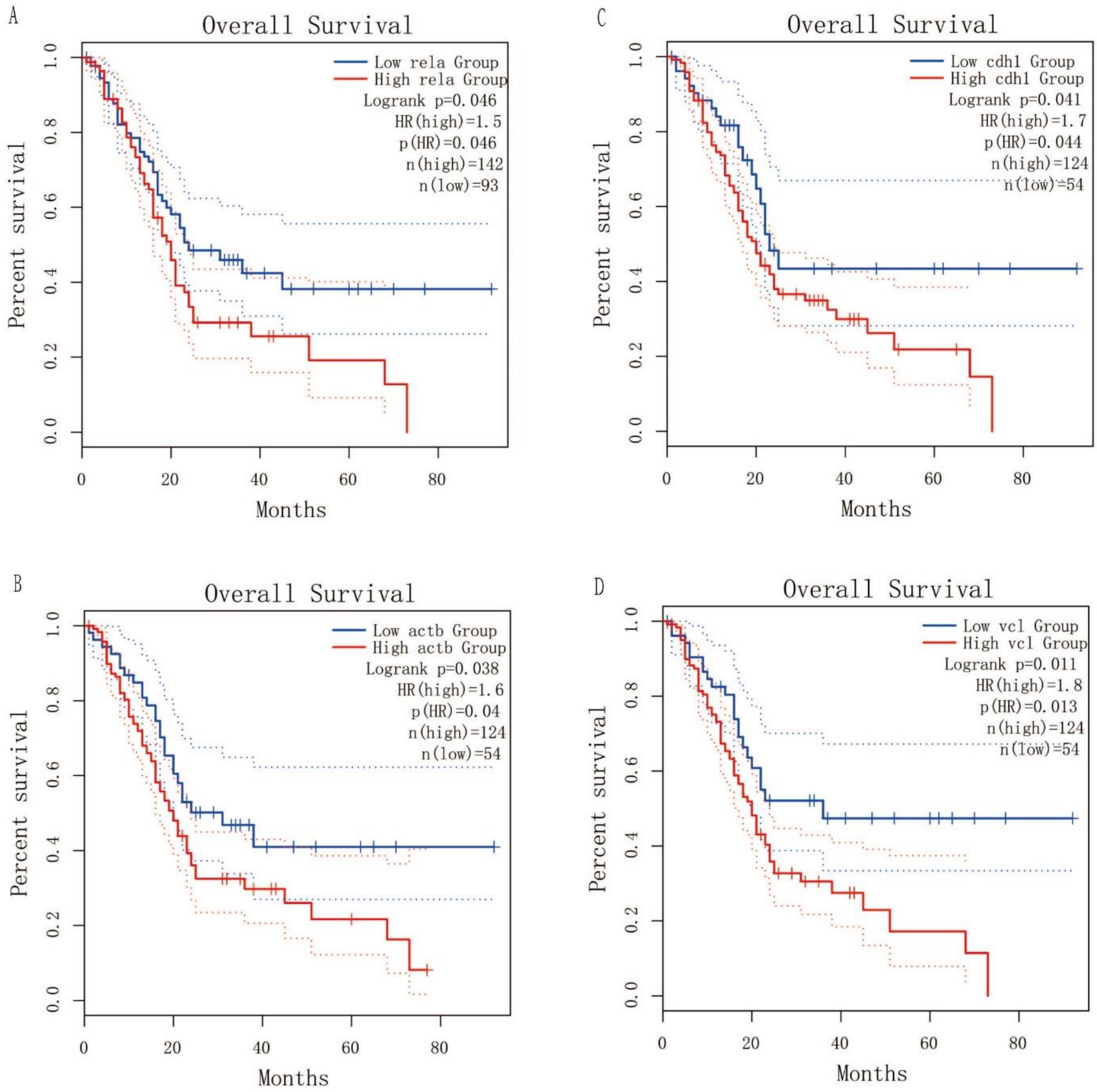

Supplementary Figure 6. Survival analysis of hub genes in pancreatic adenocarcinoma.
